# The Histone Demethylase Activity of Rph1 is Not Essential for Its Role in the Transcriptional Response to Nutrient Signaling

**DOI:** 10.1371/journal.pone.0095078

**Published:** 2014-07-07

**Authors:** Niklas Nordberg, Ida Olsson, Mattias Carlsson, Guo-Zhen Hu, Jakub Orzechowski Westholm, Hans Ronne

**Affiliations:** 1 Department of Microbiology, Swedish University of Agricultural Sciences, Uppsala, Sweden; 2 Department of Medical Biochemistry and Microbiology, Uppsala University, Uppsala, Sweden; Texas A&M University, United States of America

## Abstract

Rph1 and Gis1 are two related yeast zinc finger proteins that function as downstream effectors in the Ras/PKA, TOR and Sch9 nutrient signaling pathways. Both proteins also contain JmjC histone demethylase domains, but only Rph1 is known to be an active enzyme, demethylating lysine 36 of histone H3. We have studied to what extent the demethylase activity of Rph1 contributes to its role in nutrient signaling by performing gene expression microarray experiments on a yeast strain containing a catalytically inactive allele of *RPH1*. We find that the enzymatic activity of Rph1 is not essential for its role in growth phase dependent gene regulation. However, the ability of Rph1 to both activate and repress transcription is partially impaired in the active site mutant, indicating that the demethylase activity may enhance its function *in vivo*. Consistent with this, we find that the Rph1 mutation and a deletion of the histone H3 methylase Set2 affect the same target genes in opposite directions. Genes that are differentially expressed in the Rph1 mutant are also enriched for binding of Rpd3, a downstream effector in silencing, to their promoters. The expression of some subtelomeric genes and genes involved in sporulation and meiosis are also affected by the mutation, suggesting a role for Rph1-dependent demethylation in regulating these genes. A small set of genes are more strongly affected by the active site mutation, indicating a more pronounced role for the demethylase activity in their regulation by Rph1.

## Introduction

Eukaryotic genomes are packaged within the cell in the form of chromatin, the smallest unit of which is the nucleosome composed of 147 base pairs of DNA wrapped around an octamer of highly conserved histone proteins. Histones can be modified by phosphorylation, acetylation and methylation, with effects on many biological processes including DNA repair and transcription [1]. Histone methylation was at first thought to be a stable, irreversible modification, but proteins capable of enzymatically demethylating histones were subsequently discovered [2]–[3]. The largest group of histone demethylases is the family of proteins containing the catalytic Jumonji C (JmjC) domain, conserved among all eukaryotes [4], which is capable of enzymatically removing methyl groups from lysine residues of histones using iron and α-ketoglutarate as cofactors [5]–[6]. There has been great interest in JmjC proteins in recent years, as many of them have connections to human diseases, including cancer and neurological disorders [6]–[7].

**Figure 1:**
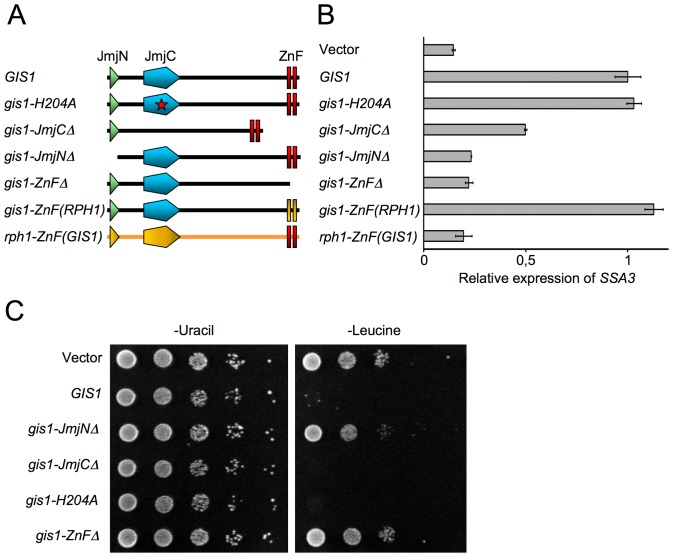
Mutational analysis of Gis1. (A) Overview of the full-length Gis1 protein and the mutant constructs that were analyzed. See Table S5 for specifics of deleted and replaced amino acids. The *gis1-ZnF(RPH1)* construct encodes the entire Gis1 protein but with its zinc fingers replaced by the zinc fingers of Rph1. Likewise, the *rph1-ZnF(GIS1)* construct encodes the entire Rph1 protein but with the zinc fingers replaced by those of Gis1. Abbreviation: ZnF, zinc fingers. (B) Expression of *SSA3* in diauxic phase *gis1*Δ yeast cells transformed with a centromeric vector (pFL38) containing various *GIS1* constructs. *SSA3* transcript levels were measured using competitive reverse transcription PCR (see Materials and Methods) and normalized against the strain containing the wild type *GIS1* construct. (C) Growth inhibition caused by overexpressing *GIS1*. Wild type yeast cells transformed with a 2-micron vector (pHR81) containing various *GIS1* constructs were spotted in 10-fold serial dilutions on synthetic uracil-less and leucine-less media and allowed to grow for 3 days before analysis. Since the pHR81 vector contains the promoter-deficient *LEU2-d* gene [63] which can only sustain growth on leucine-less media when present in a very high copy number, selection on leucine-less plates forces a higher copy number and thus also an elevated expression of the different *GIS1* constructs.

There are five JmjC domain-containing proteins in *Saccharomyces cerevisiae*, two of which are the related C_2_H_2_ zinc finger proteins Rph1 and Gis1 (Figure 1A). Rph1 is a functional histone demethylase, with the ability to reverse di- and trimethylation on lysine 36 of histone H3 (H3-K36me2 and H3-K36me3), both *in vitro* and *in vivo* [8]–[12]. This substrate of Rph1 is created by the histone methylase Set2 [13], which associates with RNA polymerase II during transcriptional elongation [14]. A downstream effector that is dependent on methylated H3-K36 is the repressive Rpd3(S) histone deacetylase (HDAC) complex, which prevents erroneous transcription initiation within open reading frames [15]–[17]. This raises the possibility that Rph1-dependent demethylation could counteract Set2 and thereby promote transcriptional elongation [7]. However, Set2-mediated H3-K36 methylation also has a role in preventing spreading of silencing from heterochromatin into neighboring euchromatic regions [18]. This “anti-silencing” function is independent of the Rpd3(S) complex, so Rph1 could potentially also function in other processes than elongation-associated regulation.

Unlike Rph1, Gis1 is likely to be an inactive enzyme due to the fact that it has a histidine-to-tyrosine substitution in one of the iron-coordinating residues of the JmjC domain [5]. In support of this view, our own work (data not shown) and that of others has failed to reveal any enzymatic activity of Gis1 when tested against all three histone lysines that are methylated in budding yeast: H3-K4 [19]–[20], H3-K36 [9], [19], and H3-K79 [19]–[20]. In one study, it was suggested that Gis1 might be capable of demethylating H3-K36me1 and H3-K36me2 [12]. However, this conclusion was based on indirect evidence such as methylation states in a *gis1*Δ strain, and these results are also compatible with a model in which Gis1 somehow modulates the activity of Rph1 or some other demethylase.

Irrespective of its possible role as a histone demethylase, Gis1 is well established as a transcription factor involved in nutrient signaling, acting downstream of the Ras/cAMP, TOR and Sch9 pathways, where it regulates genes needed for stress-resistance and long-term survival, *i.e.* resistance to chronological aging [21]–[25]. We have recently shown that Rph1 also is involved in these pathways, functioning alongside Gis1 as a repressor and activator of gene expression acting through STRE (STress Response Element) and PDS (Post-Diauxic Shift) promoter motifs [26]. A role for Rph1 in nutrient signaling is also consistent with the fact that it becomes phosphorylated after treatment with rapamycin, an inhibitor of the TOR kinase [27]. Interestingly, animal JmjC proteins have also, just like Gis1, been implicated in aging. Thus, disruption of *Drosophila* Kdm4A causes a reduced lifespan in male flies [28], and knockdown of RBR-2 in *Caenorhabditis elegans* also decreases the lifespan [29]. Mouse Ndy1/Jhdm1b counteracts cellular senescence and protects cells from oxidative stress, and this effect requires a catalytically active JmjC domain [30]–[31]. Finally, a knockdown of the human KDM8 transcript inhibits cancer cell proliferation, and this effect is also dependent on the demethylase activity of KDM8 [32].

There are some possible links between JmjC histone demethylation and nutrient signaling, the most obvious of which is that one of the cofactors needed for the demethylation reaction is α-ketoglutarate, which is converted to succinate in this reaction. Succinate and α-ketoglutarate are both intermediates in the TCA cycle, and it is thus possible that the energy status of the cell could affect JmjC-catalyzed demethylation through the availability of α-ketoglutarate [33]. Consistent with this, the human JmjC demethylase KDM2A represses transcription of rRNA in response to starvation, a process that is dependent on JmjC catalytic activity and is inhibited by succinate [34]. Recent evidence suggests that Rph1 also functions in chronological aging, though in a different way than Gis1. Thus, while Gis1 acts redundantly with Msn2 and Msn4 in promoting longevity under standard growth conditions, Rph1 seems to mediate longevity extension in response to oxidative stress induced by the drug menadione. Furthermore, it was proposed that this effect involves Rph1-dependent demethylation of histones on subtelomeric genes [35].

In summary, Rph1 and Gis1 function together as downstream effectors in nutrient signaling, by binding to STRE and PDS motifs in the promoters of target genes [26]. In addition, Rph1 is a histone demethylase, while Gis1 is most likely an inactive enzyme. Furthermore, recent reports have revealed that the JmjC domain of Gis1, whether active or inactive, is dispensable for its role as a transcriptional activator [36]–[37]. In contrast, the demethylase activity of Rph1 affects its ability to repress the DNA damage repair gene *PHR1* [38], but it is not known if this activity is also needed for the growth phase-dependent gene regulation that is mediated by Rph1 [26]. We decided to address this gap of knowledge by transcriptional profiling of a yeast strain carrying an allele of Rph1 that has a catalytically inactive JmjC domain, and find that an active JmjC domain is largely dispensable for the role of Rph1 in nutrient signaling. However, catalytic inactivation of Rph1 does have subtle effects on subtelomeric gene expression, which is consistent with a model where the enzymatic activity of Rph1 opposes Set2-dependent gene regulation.

## Results

### Deletion, point mutation and domain swap analysis of Gis1 function

In addition to its two DNA binding zinc fingers, which mediate binding to the PDS and STRE promoter motifs [39]–[40], Gis1 also contains JmjN and JmjC domains [4]. However, Gis1 is likely an inactive enzyme since it has a histidine-to-tyrosine substitution in one of the iron-binding residues of the JmjC domain [5]. Consistent with this, we were neither able to detect any *in vitro* histone demethylase activity in purified Gis1 protein, nor did we detect any alterations of global methylation in *gis1*Δ cells using Western blots with methylation-specific antibodies (data not shown). Others have similarly failed to detect any demethylase activity associated with Gis1 [9], [19]–[20].

Since it is hard to prove that a protein lacks enzymatic activity, we also tested the effect of a mutation that would render any JmjC type demethylase inactive. For this, we chose histidine 204. This histidine is predicted to bind iron, a cofactor of the enzyme [5], [41], and mutating it abolishes histone demethylase activity both in Rph1 [8]–[10], [12] and in several mammalian JmjC proteins [42]–[45]. The resulting *gis1-H204A* mutant was expressed from a plasmid in a *gis1*Δ strain. As a reporter for nutrient signaling, we used the *SSA3* gene, which is strongly induced in a Gis1-dependent manner during the diauxic shift. We found that the *gis1-H204A* mutation had no significant effect on *SSA3* induction (Figure 1B). This suggests that the role of Gis1 in regulating *SSA3* is independent of any demethylase activity that might be present in Gis1. We also tested a deletion of the entire Gis1 JmjC domain. This protein can still mediate *SSA3* induction, though not as efficiently as the wild type protein (Figure 1B). In contrast, a deletion of the JmjN domain rendered the construct inactive (Figure 1B). It should be noted that since Gis1 is regulated by proteolysis [37], the reduced or absent transcriptional activity of these constructs could be due to increased proteolysis. However, the fact that Gis1 is partially active without the JmjC domain provides further strong evidence that any activity provided by this domain is dispensable for *SSA3* induction. These findings are consistent with the results of others [36]–[37].

We proceeded to test the effect of deleting the DNA-binding zinc fingers in Gis1. As shown in Figure 1B, the resulting construct is completely inactive. This is not surprising since the zinc fingers are needed for binding to the *SSA3* promoter. What is more interesting is the result of a domain swap experiment where we put the Rph1 zinc fingers into Gis1, and conversely the Gis1 zinc fingers into Rph1. We found that Gis1 with zinc fingers from Rph1 is as active as wild type Gis1, whereas Rph1 with zinc fingers from Gis1 is inactive (Figure 1B). This shows that the few differences that exist between Gis1 and Rph1 in the zinc fingers do not explain the specific regulation of *SSA3* by Gis1 but not by Rph1. The question then arises why *SSA3* is regulated only by Gis1, when the zinc fingers of Rph1 also can mediate this regulation. There are at least two possible explanations. One is that both Gis1 and Rph1 can bind to the *SSA3* promoter *in vivo*, but only Gis1 is able to activate transcription. Against this hypothesis argues the fact that both Gis1 and Rph1 are active as transcriptional activators during the diauxic shift, but largely on non-overlapping target genes [26]. Another possible explanation is if Gis1 (and also Rph1) needs something more than just the binding to a target promoter to activate it. It is therefore interesting to note that we previously saw evidence of context-dependent gene regulation by Gis1 and Rph1 [26].

We further tested the effect of overexpressing the different Gis1 constructs by using selection for the promoter-deficient *LEU2-d* gene which forces the plasmid to a high copy number. In agreement with previous data [22], [36]–[37], we found that overexpression of *GIS1* inhibits growth (Figure 1C). Just like the ability to activate *SSA3* (Figure 1B), this effect is blocked by deletion of either the zinc fingers or the JmjN domain, but not by a deletion of the JmjC domain. We note that overexpression of *RPH1* also inhibits growth and, just as for *GIS1*, this is dependent on the zinc fingers, but not on the JmjC domain [10]. Taken together, it is clear that neither the ability of Gis1 to activate genes, at least the hallmark target gene *SSA3*, nor its ability to inhibit growth when overexpressed is dependent on its JmjC domain. However, this is not surprising if Gis1 is an inactive enzyme (see above). In contrast, Rph1, which we have shown to act alongside Gis1 in growth phase-dependent control of gene expression [26], is an active demethylase. We therefore proceeded to investigate what role the enzymatic activity of Rph1 plays in gene regulation.

### Effects of a catalytic site point mutation in Rph1

To identify genes whose regulation depend on the histone demethylase activity of Rph1, we used gene expression microarrays on a yeast strain where the catalytic site of the Rph1 JmjC domain carries the same histidine to alanine substitution as we used in Gis1 (H235A in Rph1), known to render Rph1 enzymatically inactive [8]–[10], [12]. As controls we used the parental wild type (WT) strain and the knockout mutant *rph1*Δ. We reasoned that if the catalytic activity of Rph1 is essential for its role as a transcription factor, the *rph1-H235A* mutant should behave more similar to the *rph1*Δ knockout, whereas it should behave more similar to the wild type if transcriptional regulation is independent of the histone demethylase activity.

A major finding of our previous work [26] was that Rph1 regulates many of the same genes as its homolog Gis1, but the interplay between the two transcription factors is quite complex, being synergistic, redundant or antagonistic. For this reason we also investigated the role of the *rph1-H235A* mutant in a strain where *GIS1* had been deleted. Furthermore, since the genes that are regulated by Rph1 and/or Gis1 differ depending on the growth phase [26], we performed the analysis at two time points: in exponential growth (log phase), and shortly after the cells have run out of glucose and switched to respiratory growth (PDS phase).

**Figure 2:**
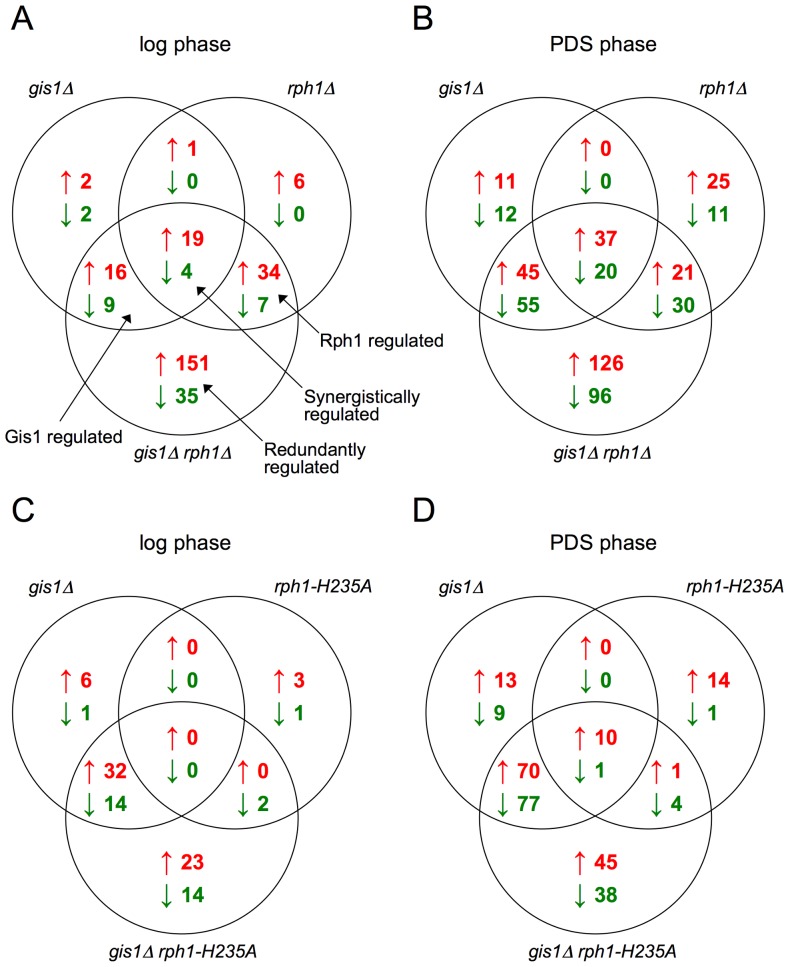
Gene expression changes in response to deletions or mutations of *GIS1* and *RPH1*. Genes significantly upregulated (red) or downregulated (green) in the *gis1*Δ vs. WT, *rph1*Δ vs. WT and *gis1*Δ *rph1*Δ vs. WT comparisons during log phase (A) and PDS phase (B), and genes significantly affected in the *gis1*Δ vs. WT, *rph1-H235A* vs. WT and *gis1Δ rph1-H235A* vs. WT during log phase (C) and PDS phase (D).

After the transcriptional profiling of the six strains (WT, *rph1-H235A*, *rph1*Δ, *gis1*Δ, *gis1*Δ *rph1-H235A* and *gis1*Δ *rph1*Δ) had been carried out, we searched for genes whose expression differ significantly in a pairwise comparison of any two strains. By looking first at only the WT and the full knockout strains (*gis1*Δ, *rph1*Δ, and *gis1*Δ *rph1*Δ), it is clear that Gis1 and Rph1 both repress and activate gene expression, as many genes are affected by the knockouts (Figure 2A–B). The patterns agree with our earlier findings [26] that Gis1 and Rph1 mainly act as redundant repressors in the log phase (Figure 2A), but function as both repressors and activators in the PDS phase, targeting partly overlapping sets of genes (Figure 2B).

**Figure 3:**
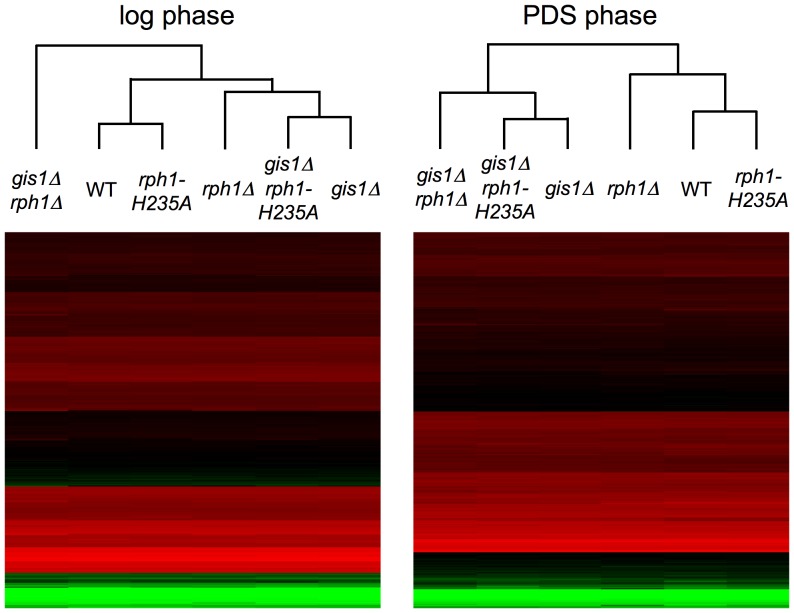
Hierarchical clustering of the whole genome expression profiles for the six strains under study. Separate clusterings were made in log phase and PDS phase since growth phase was the most heavily weighted factor contributing to the expression patterns.

Compared to the substantial effects on gene expression that were seen in the *rph1*Δ knockout, the effects of the *rph1-H235A* mutation were much more limited. In the clustering, which takes into account the expression levels of all genes, the *rph1-H235A* strain was much more similar to the wild type than to the *rph1*Δ strain, and the profile of the *gis1*Δ *rph1-H235A* strain resembled that of *gis1*Δ rather than of *gis1*Δ *rph1*Δ (Figure 3). This pattern was evident also when looking at the number of individual genes that were significantly affected in each strain (Table 1, Figure 2C–D). For example, with the thresholds we used, 60 genes are significantly upregulated in the log phase when *RPH1* is deleted, but only three genes go up in the *rph1-H235A* mutant. We conclude that most of the transcriptional regulation that is mediated by Rph1 is independent of its demethylase activity. The only apparent exception are genes that are upregulated in the PDS phase, where as many as 25 genes are significantly affected by the point mutation (Table 1, Figure 2D). Interestingly, this effect is not seen in the *gis1*Δ background where not a single additional gene is upregulated when the *rph1-H235A* mutation also is present.

**Table 1:** Numbers of differentially expressed genes in different strain comparisons.

	Log phase		PDS phase	
Comparison	Up	Down	Up	Down
*rph1-H235A* vs. WT	3	3	25	6
*rph1*Δ vs. WT	60	11	83	61
*gis1Δ rph1-H235A* vs. *gis1*Δ	2	4	0	9
*gis1*Δ *rph1*Δ vs. *gis1*Δ	162	27	46	38
*gis1*Δ vs. WT	38	15	93	87
*gis1Δ rph1-H235A* vs. WT	55	30	126	120
*gis1*Δ *rph1*Δ vs. WT	220	55	229	201

Genes exhibiting 1.5-fold differential expression at p<0.01 are included.

**Figure 4:**
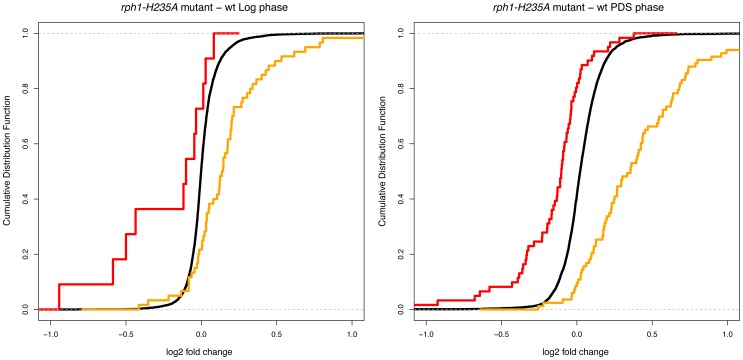
Cumulative distribution functions for the effects of the *rph1-H235A* mutation on gene expression. The genes examined in our arrays were divided into three groups: those that were significantly downregulated in the *rph1*Δ knockout (*red*), those that were significantly upregulated in the *rph1*Δ knockout (*yellow*), and all other genes (*black*). The graph to the *left* shows the effects in log phase and the graph to the *right* in PDS phase.

Since the number of genes affected are determined by the thresholds that we used (>1.5-fold change, p<0.01) we wondered if the *rph1-H235A* mutant might have more subtle effects on the genes that were significantly affected by the *rph1*Δ knockout. We therefore examined the genes significantly affected by the *rph1*Δ knockout under each condition as a group, and tested if they were significantly up- or downregulated, compared to all other genes in the genome. We found that the groups of genes that are affected by the *rph1*Δ knockout are affected in the same direction by the *rph1-H235A* mutation, though the effects are smaller and therefore fall below our significance thresholds for individual genes (Table 2). The effect was significant (p<0.01) for all groups of genes. The effect was still seen when the few genes significantly affected by the *rph1-H235A* mutation were removed, so it is not due to the presence of these genes within the group (Table 2). To further examine this effect, we made cumulative distribution plots in which the effects of the *rph1-H235A* mutation on expression of genes that were either significantly upregulated or downregulated in the *rph1*Δ knockout was compared to all other genes (Figure 4). These plots confirm that the effect is not limited to a small subset of genes. It thus seems that the *rph1-H235A* mutation has an effect on the genes that are regulated by Rph1 in response to nutrient signaling, but the effect is for most genes much smaller than the effect of the *rph1*Δ knockout. We conclude that the histone demethylase activity of Rph1 is not essential for regulation of most target genes by Rph1. The fact that the *rph1-H235A* mutation does have a small effect on the expression of many Rph1-regulated genes could mean that the catalytic activity enhances the gene regulatory function of Rph1 or that the mutation affects the folding or stability of Rph1.

**Figure 5:**
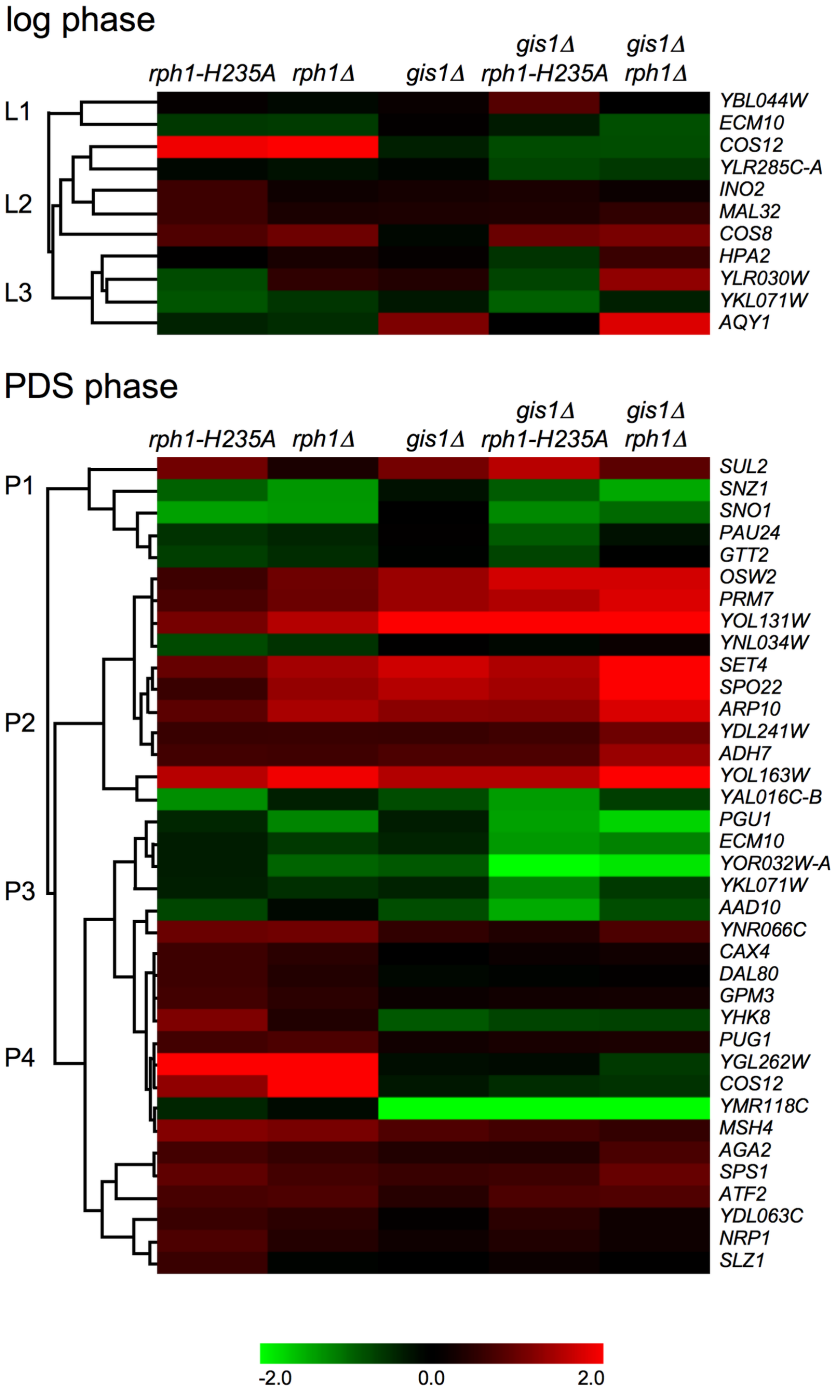
Hierarchical clustering of expression profiles for genes specifically affected by the *rph1-H235A* mutation. Genes that had a significant differential gene expression in any of the *rph1-H235A* comparisons were clustered with regards to how they were affected by the *rph1*Δ and *gis1*Δ knockouts. Separate clusterings were made in log phase and PDS phase since the affected genes in each growth phase are largely non-overlapping. In all cases, the heat maps show log ratios of differential gene expression compared to the wild type strain.

**Table 2:** Effect of the *rph1-H235A* mutation on genes regulated by Rph1.

All genes significantly affected (1.5-fold, p<0.01) by the *rph1*Δ knockout							
Up in log (60)		Down in log (11)		Up in PDS (83)		Down in PDS (61)	
Fold	p-value	Fold	p-value	Fold	p-value	Fold	p-value
**↑ 1.13**	**3.0E-10**	**↓ 0.86**	**4.4E-3**	**↑ 1.30**	**<2E-16**	**↓ 0.87**	**1.5E-13**

Genes significantly affected also by the *rph1-H235A* mutation removed							
Up in log (59)		Down in log (10)		Up in PDS (68)		Down in PDS (59)	
Fold	p-value	Fold	p-value	Fold	p-value	Fold	p-value
**↑ 1.10**	**8.9E-10**	**↓ 0.88**	**1.5E-2**	**↑ 1.19**	**<2E-16**	**↓ 0.89**	**1.5E-12**

The groups of genes that were significantly affected in the *rph1*Δ knockout (row 2 in Table 1) were analyzed for their response to the *rph1-H235A* mutation. Expression of each group of genes was tested against the rest of the genome with a Wilcoxon rank sum test. The mean fold change is shown, with p-values<0.02 highlighted in bold, and with arrows denoting up- and downregulation. *Top:* all genes that were significantly affected by the *rph1*Δ knockout were included. *Bottom:* only those genes not significantly affected (on their own) by the *rph1-H235A* mutation were included.

We also considered the possibility that Rph1 might have global effects on gene expression that would depend on its demethylase activity. One possibility would be that Rph1 affects all transcription, perhaps by promoting elongation and thereby slightly upregulating all genes, as suggested by Kim and Buratowski [9]. This would not be detected in our analysis since such global effects are precisely what the normalization strives to eliminate. We therefore carried out an analysis of unnormalized data using the scheme devised by Takahashi et al. [46]. This analysis did not reveal any significant global effects on gene expression (data not shown). We conclude that the *rph1-H235A* mutation does not cause any major global effects on gene expression, at least not under the conditions studied by us, but more subtle effects on large sets of genes are still possible.

A total of 45 genes were significantly affected by the *rph1-H235A* mutation in at least one of the two growth phases, *i.e.* they change significantly in either the *rph1-H235A* vs. WT or the *gis1*Δ *rph1-H235A* vs. *gis1*Δ comparison (Tables S1 and S2). There were several patterns of regulation. To further analyze these patterns, and elucidate which genes are similarly affected by the *rph1-H235A* mutation, we carried out hierarchical clustering of the expression profiles (Figure 5). The genes were clustered separately for log phase (11 genes) and PDS phase (37 genes). The effect of the *rph1-H235A* mutation on gene expression in PDS phase was verified by qPCR for two of the genes: *COS12* and *YOL131W* (Table S3).

### Overlap between Rph1-regulated and Set2-regulated genes

The target of Rph1 demethylation is di- and trimethylated H3-K36 [8]–[10], [12], a mark laid down by Set2 [13]. Tompa and Madhani [18] found that 290 genes are significantly downregulated and 492 genes significantly upregulated in a *set2*Δ strain. In agreement with the notion that Rph1 demethylates H3-K36, and thus counteracts Set2, we found that these groups of genes are significantly affected by the *rph1-H235A* point mutation, and in the opposite direction from the effect of *set2*Δ (Table 3). However, the effects of the *rph1* mutation on individual genes are small, and hence only a few genes pass the thresholds for being included in Figure 5 and Tables S1 and S2. Still, when considered as a group, the Set2 targets are affected by the *rph1-H235A* mutation to a high statistical significance. Interestingly, Gis1 also seems to regulate this set of genes in log phase.

**Table 3:** Differential expression of genes affected by the *set2*Δ deletion.

	Down in *set2*Δ(a)		Up in *set2*Δ(b)	
Log phase	Fold	p-value	Fold	p-value
*rph1-H235A* vs. WT	**↑ 1.06**	**<2E-16**	**↓ 0.97**	**2.5E-13**
*rph1*Δ vs. WT	**↑ 1.04**	**4.4E-07**	**↑ 1.01**	**2.4E-03**
*gis1*Δ *rph1-H235A* vs. *gis1*Δ	**↑ 1.02**	**2.5E-04**	**↑ 1.01**	**2.1E-03**
*gis1*Δ *rph1*Δ vs. *gis1*Δ	**↑ 1.03**	**0.03**	**↑ 1.07**	**<2E-16**
*gis1*Δ vs. WT	**↑ 1.06**	**<2E-16**	**↓ 0.97**	**7.0E-04**
*gis1*Δ *rph1-H235A* vs. WT	**↑ 1.07**	**<2E-16**	0.98	0.36
*gis1*Δ *rph1*Δ vs. WT	**↑ 1.09**	**8.2E-13**	**↑ 1.04**	**1.1E-05**

PDS phase	Fold	p-value	Fold	p-value
*rph1-H235A* vs. WT	**↑ 1.05**	**2.0E-12**	**↓ 0.98**	**2.1E-06**
*rph1*Δ vs. WT	**↑ 1.05**	**3.6E-05**	**↓ 0.97**	**4.2E-05**
*gis1*Δ *rph1-H235A* vs. *gis1*Δ	**↑ 1.02**	**7.2E-03**	**↓ 0.98**	**4.8E-08**
*gis1*Δ *rph1*Δ vs. *gis1*Δ	**↑ 1.05**	**5.2E-05**	0.99	0.47
*gis1*Δ vs. WT	1.00	0.29	0.99	0.39
*gis1*Δ *rph1-H235A* vs. WT	1.02	0.84	0.97	0.16
*gis1*Δ *rph1*Δ vs. WT	1.05	0.27	0.98	0.89

(a) The group of 290 genes significantly downregulated in *set2*Δ [18].

(b) The group of 492 genes significantly upregulated in *set2*Δ [18].

Expression of these groups of genes was tested against the rest of the genome with a Wilcoxon rank sum test. The mean fold change is shown, with p-values<0.05 highlighted in bold, and with arrows denoting up- and downregulation.

Set2-mediated H3-K36 methylation promotes histone deacetylation by the Rpd3(S) complex, which prevents erroneous transcription initiation within open reading frames [15]–[17]. The Rpd3(S) complex has five subunits: Rpd3, Sin3, Ume1, Eaf3 and Rco1. The first three are shared with the Rpd3(L) complex whereas Eaf3 and Rco1 are specific for Rpd3(S) and mediate its interaction with methylated H3-K36 [15]–[17], [47]. As shown in Tables 4 and 5, we found a certain degree of anti-correlation between genes that are regulated by Rph1 and those that are regulated by Rco1 and/or Eaf3, similar to the anti-correlation between genes regulated by Set2 and Rph1. Thus, the effect of the *rph1-H235A* mutation or the *rph1*Δ deletion, when significant, was always the opposite of the *rco1*Δ or *eaf3*Δ deletions. However, most of these effects were only marginally significant, and were seen in only some comparisons, that with one exception were restricted to the log phase.

**Table 4:** Differential expression of genes downregulated by deletion of subunits of the Rpd3(S) and Rpd3(L) complexes.

					Rpd3(L)		Rpd3(L)		Rpd3(L)		Rpd3(L)	
	Rpd3(S)		Rpd3(S)		Rpd3(S)		Rpd3(S)					
	Down in *eaf3*Δ (27)		Down in *rco1*Δ (106)		Down in *rpd3*Δ (105)		Down in *sin3*Δ (122)		Down in *dep1*Δ (121)		Down in *pho23*Δ (111)	
Log phase	Fold	p-value	Fold	p-value	Fold	p-value	Fold	p-value	Fold	p-value	Fold	p-value
*rph1-H235A* vs. WT	1.03	0.40	**↑ 1.03**	**0.04**	**↑ 1.02**	**6.7E-03**	**↑ 1.04**	**0.03**	1.02	0.86	1.01	0.35
*rph1*Δ vs. WT	1.10	0.18	1.06	0.15	**↑ 1.08**	**4.6E-03**	1.06	0.41	1.07	0.70	1.04	0.28
*gis1*Δ *rph1-H235A* vs. *gis1*Δ	**↑ 1.05**	**0.02**	0.99	0.78	0.99	0.72	1.00	1.00	1.01	0.92	1.02	0.94
*gis1*Δ *rph1*Δ vs. *gis1*Δ	**↑ 1.14**	**0.04**	**↑ 1.08**	**0.03**	**↑ 1.07**	**0.05**	1.05	0.18	**↑ 1.11**	**0.05**	**↑ 1.13**	**2.5E-03**
*gis1*Δ vs. WT	1.06	0.75	1.01	0.32	**↑ 1.07**	**0.02**	1.03	0.52	1.03	0.77	1.05	0.32
*gis1*Δ *rph1-H235A* vs. WT	1.12	0.10	1.00	0.67	1.06	0.14	1.03	0.86	1.05	0.68	1.07	0.08
*gis1*Δ *rph1*Δ vs. WT	1.21	0.09	**↑ 1.09**	**0.01**	**↑ 1.15**	**7.7E-03**	1.08	0.32	1.15	0.25	**↑ 1.19**	**3.5E-03**

PDS phase	Fold	p-value	Fold	p-value	Fold	p-value	Fold	p-value	Fold	p-value	Fold	p-value
*rph1-H235A* vs. WT	1.00	0.59	**↑ 1.05**	**0.03**	1.03	0.15	1.03	0.19	1.02	0.16	1.04	0.04
*rph1*Δ vs. WT	0.99	0.49	1.07	0.29	**↑ 1.08**	**7.2E-03**	1.08	0.11	**↑ 1.11**	**1.4E-03**	**↑ 1.11**	**6.5E-03**
*gis1*Δ *rph1-H235A* vs. *gis1*Δ	0.99	0.57	0.98	0.11	**↓ 0.99**	**0.01**	1.00	0.10	**↓ 0.98**	**0.05**	0.99	0.09
*gis1*Δ *rph1*Δ vs. *gis1*Δ	1.04	0.07	1.01	0.89	**↑ 1.08**	**2.0E-03**	**↑ 1.07**	**2.9E-03**	**↑ 1.09**	**1.4E-03**	**↑ 1.11**	**9.4E-05**
*gis1*Δ vs. WT	0.89	0.78	1.01	0.68	**↑ 1.08**	**2.2E-03**	1.05	0.09	**↑ 1.06**	**0.03**	**↑ 1.12**	**1.9E-03**
*gis1*Δ *rph1-H235A* vs. WT	0.89	0.88	0.98	0.46	**↑ 1.07**	**0.04**	1.05	0.14	1.05	0.09	**↑ 1.11**	**0.01**
*gis1*Δ *rph1*Δ vs. WT	0.93	0.20	1.00	0.74	**↑ 1.17**	**3.4E-04**	**↑ 1.13**	**2.4E-03**	**↑ 1.15**	**2.0E-03**	**↑ 1.24**	**6.9E-05**

Shown are the groups of genes downregulated 2-fold or more in the indicated deletion strains [17]. The number of genes in these groups is noted in parenthesis. Expression of these groups of genes was tested against the rest of the genome with a Wilcoxon rank sum test. The mean fold change is shown, with p-values<0.05 highlighted in bold, and with arrows denoting up- and downregulation.

**Table 5:** Differential expression of genes upregulated by deletion of subunits of the Rpd3(S) and Rpd3(L) complexes.

					Rpd3(L)		Rpd3(L)		Rpd3(L)		Rpd3(L)	
	Rpd3(S)		Rpd3(S)		Rpd3(S)		Rpd3(S)					
	Up in *eaf3*Δ (41)		Up in *rco1*Δ (116)		Up in *rpd3*Δ (110)		Up in *sin3*Δ (130)		Up in *dep1*Δ (141)		Up in *pho23*Δ (99)	
Log phase	Fold	p-value	Fold	p-value	Fold	p-value	Fold	p-value	Fold	p-value	Fold	p-value
*rph1-H235A* vs. WT	1.00	0.92	**↓ 0.97**	**0.01**	1.01	0.60	1.00	0.88	1.00	0.24	1.01	0.32
*rph1*Δ vs. WT	0.97	0.43	0.97	0.10	**↑ 1.06**	**1.2E-03**	**↑ 1.06**	**3.0E-03**	**↑ 1.08**	**1.3E-03**	**↑ 1.08**	**5.7E-06**
*gis1*Δ *rph1-H235A* vs. *gis1*Δ	1.02	0.11	**↓ 0.96**	**0.02**	1.01	0.16	**↑ 1.02**	**0.02**	**↑ 1.04**	**0.02**	**↑ 1.03**	**2.4E-03**
*gis1*Δ *rph1*Δ vs. *gis1*Δ	0.95	0.75	1.02	0.60	**↑ 1.12**	**7.3E-05**	**↑ 1.15**	**5.4E-07**	**↑ 1.23**	**1.5E-08**	**↑ 1.16**	**4.7E-08**
*gis1*Δ vs. WT	**↓ 0.94**	**0.04**	0.99	0.36	**↑ 1.04**	**5.8E-03**	0.99	0.86	1.00	0.49	1.00	0.93
*gis1*Δ *rph1-H235A* vs. WT	0.96	0.58	**↓ 0.95**	**0.01**	**↑ 1.05**	**0.01**	1.01	0.51	1.04	0.18	**↑ 1.04**	**0.04**
*gis1*Δ *rph1*Δ vs. WT	0.90	0.74	1.01	0.59	**↑ 1.17**	**8.6E-06**	**↑ 1.15**	**1.5E-04**	**↑ 1.23**	**8.5E-06**	**↑ 1.16**	**6.5E-07**

PDS phase	Fold	p-value	Fold	p-value	Fold	p-value	Fold	p-value	Fold	p-value	Fold	p-value
*rph1-H235A* vs. WT	1.01	0.22	0.98	0.43	0.98	0.20	1.01	0.25	0.99	0.09	0.99	0.92
*rph1*Δ vs. WT	1.02	0.48	0.97	0.09	1.04	0.30	**↑ 1.09**	**3.3E-03**	1.04	0.29	1.06	0.32
*gis1*Δ *rph1-H235A* vs. *gis1*Δ	0.98	0.16	0.97	0.14	0.99	0.34	1.00	0.44	0.99	0.32	1.01	0.92
*gis1*Δ *rph1*Δ vs. *gis1*Δ	0.97	0.81	0.99	0.45	**↑ 1.05**	**1.7E-04**	**↑ 1.05**	**7.4E-03**	**↑ 1.04**	**8.9E-03**	**↑ 1.06**	**0.02**
*gis1*Δ vs. WT	1.01	0.79	1.00	0.59	1.07	0.06	1.06	0.28	1.02	0.85	0.99	0.31
*gis1*Δ *rph1-H235A* vs. WT	1.00	0.74	0.98	0.10	1.05	0.56	1.06	0.40	1.00	0.70	0.99	0.20
*gis1*Δ *rph1*Δ vs. WT	0.98	0.70	0.99	0.39	**↑ 1.12**	**6.3E-04**	**↑ 1.11**	**0.02**	1.05	0.39	1.05	0.59

Shown are the groups of genes upregulated 2-fold or more in the indicated deletion strains [17]. The number of genes in these groups is noted in parenthesis. Expression of these groups of genes was tested against the rest of the genome with a Wilcoxon rank sum test. The mean fold change is shown, with p-values<0.05 highlighted in bold, and with arrows denoting up- and downregulation.

In contrast, we saw a much stronger correlation between genes regulated by Rph1 and/or Gis1 and those that are regulated by Rpd3 and Sin3, which are subunits of both Rpd3(S) and Rpd3(L), as well as Dep1 and Pho23, which are specific to the Rpd3(L) complex (Tables 4 and 5). This suggests that Rpd3(L) plays a more important role than Rpd3(S) in regulating those genes that are also regulated by Rph1 and/or Gis1. Surprisingly, these genes were in general upregulated in the *rph1*Δ and *gis1*Δ strains, regardless of whether they were upregulated or downregulated by deletion of the Rpd3(L) subunits. In contrast to the genes affected by the *eaf3*Δ or *rco1*Δ deletions, where most of the significant effects involved Rph1, the genes regulated by Rpd3(L) appear to be synergistically repressed by Gis1 and Rph1, with little to no effect of *rph1-H235A*. As seen in Table 6, an even stronger correlation is seen between genes that are regulated by Gis1 and/or Rph1 and genes known to bind Rpd3 in their promoters, but not within the open reading frame [48]. This is also consistent with the Rpd3(L) complex being involved as it acts through promoter binding while the Rpd3(S) complex is active within the transcribed region. Also in this case, the genes were generally upregulated in the *rph1*Δ and *gis1*Δ strains.

**Table 6:** Differential expression of genes that bind the Rpd3 protein.

	Promoter binding(a)		ORF binding(b)	
Log phase	Fold	p-value	Fold	p-value
*rph1-H235A* vs. WT	**↑ 1.03**	**2.4E-13**	1.01	0.18
*rph1*Δ vs. WT	**↑ 1.02**	**<2E-16**	1.02	0.34
*gis1*Δ *rph1-H235A* vs. *gis1*Δ	1.00	0.20	1.01	0.47
*gis1*Δ *rph1*Δ vs. *gis1*Δ	**↓ 0.99**	**0.02**	0.99	0.62
*gis1*Δ vs. WT	**↑ 1.04**	**<2E-16**	1.01	0.19
*gis1*Δ *rph1-H235A* vs. WT	**↑ 1.04**	**<2E-16**	1.02	0.10
*gis1*Δ *rph1*Δ vs. WT	**↑ 1.03**	**2.4E-15**	1.00	0.97

PDS phase	Fold	p-value	Fold	p-value
*rph1-H235A* vs. WT	**↑ 1.01**	**2.3E-04**	1.00	0.72
*rph1*Δ vs. WT	**↑ 1.04**	**5.0E-11**	1.01	0.46
*gis1*Δ *rph1-H235A* vs. *gis1*Δ	**↑ 1.04**	**<2E-16**	1.00	0.59
*gis1*Δ *rph1*Δ vs. *gis1*Δ	**↓ 0.99**	**0.05**	1.01	0.57
*gis1*Δ vs. WT	1.00	0.70	1.01	0.35
*gis1*Δ *rph1-H235A* vs. WT	**↑ 1.03**	**1.6E-05**	1.01	0.25
*gis1*Δ *rph1*Δ vs. WT	0.99	0.08	1.02	0.31

(a) The group of 595 genes whose promoters are enriched for Rpd3 binding at 2.5-fold or more [48].

(b) The group of 292 genes whose coding regions are enriched for Rpd3 binding at 1.5-fold or more [48].

Expression of these groups of genes was tested against the rest of the genome with a Wilcoxon rank sum test. The mean fold change is shown, with p-values<0.05 highlighted in bold, and with arrows denoting up- and downregulation.

We conclude that there are significant correlations between genes that are regulated by Rph1 and/or Gis1 and those that are regulated by Rpd3, indicating a possible role for the latter in Rph1- and Gis1-dependent gene regulation. However, the correlations are complex, and suggest that at least two mechanisms may be involved. One mechanism has a more limited effect, acts primarily through Rph1, is dependent on its demethylase activity, and may involve the Rpd3(S) complex. This interaction is antagonistic in that a deletion or mutation of Rph1 has the opposite effect of deleting Rpd3(S) subunits, which is in agreement with the idea that demethylation of H3-K36 reduces activation of Rpd3(S). The second mechanism has a more pronounced effect, and involves genes that are repressed by both Gis1 and Rph1, in log phase as well as in the diauxic phase. The fact that these genes are either up- or downregulated by loss of Rpd3(L) subunits suggests that Rpd3(L) does not simply act downstream of Gis1 and Rph1 even if it regulates the same set of genes. In this context, we note that Rpd3(L) has, similarly to Gis1 and Rph1, been implicated in both TOR signaling [49]–[50] and the stress response [51].

### Effects of Rph1 on the expression of subtelomeric genes

Genes that are activated by Set2, and thus downregulated in a *set2*Δ strain, are enriched close to telomeres [18]. Set2 and H3-K36 methylation have thus been identified as “anti-silencing” factors for these genes: in their absence Sir-mediated silencing spreads from heterochromatic regions into euchromatin. The mechanism is not understood, but it is not mediated by the Rpd3(S) complex as deletion of *EAF3* and *RCO1* does not cause ectopic silencing [18]. Interestingly, a substantial fraction (6 of 45) of the genes whose expression is significantly affected by the *rph1-H235A* mutation is located within 10 kb of the telomeric repeats, and 14 of 45 genes are located within 30 kb of the repeats. Furthermore, we found that deleting or mutating *RPH1* significantly increases PDS phase expression of genes that are located within 20 kb of the telomeres, as compared to the rest of the genome (Table 7, the *rph1-H235A* vs. WT and *rph1*Δ vs. WT comparisons). Similar though less pronounced effects were also in log phase (Table 7).

**Table 7:** Differential expression of subtelomeric genes.

	Subtelomeric genes(a)	
Log phase	Fold	p-value
*rph1-H235A* vs. WT	**↑ 1.02**	**4.4E-03**
*rph1*Δ vs. WT	1.04	0.30
*gis1*Δ *rph1-H235A* vs. *gis1*Δ	0.97	0.05
*gis1*Δ *rph1*Δ vs. *gis1*Δ	**↑ 1.05**	**0.01**
*gis1*Δ vs. WT	1.00	0.30
*gis1*Δ *rph1-H235A* vs. WT	0.97	0.16
*gis1*Δ *rph1*Δ vs. WT	**↑ 1.05**	**0.03**

PDS phase	Fold	p-value
*rph1-H235A* vs. WT	**↑ 1.06**	**1.1E-05**
*rph1*Δ vs. WT	**↑ 1.08**	**4.0E-03**
*gis1*Δ *rph1-H235A* vs. *gis1*Δ	**↓ 0.97**	**1.6E-04**
*gis1*Δ *rph1*Δ vs. *gis1*Δ	**↑ 1.06**	**5.6E-05**
*gis1*Δ vs. WT	**↑ 1.04**	**5.9E-03**
*gis1*Δ *rph1-H235A* vs. WT	1.01	0.21
*gis1*Δ *rph1*Δ vs. WT	**↑ 1.11**	**2.9E-05**

(a) The group of 221 genes located within 20 kb of a chromosomal end. Expression of this group of genes was tested against the rest of the genome with a Wilcoxon rank sum test. The mean fold change is shown, with p-values<0.05 highlighted in bold, and with arrows denoting up- and downregulation.

**Figure 6:**
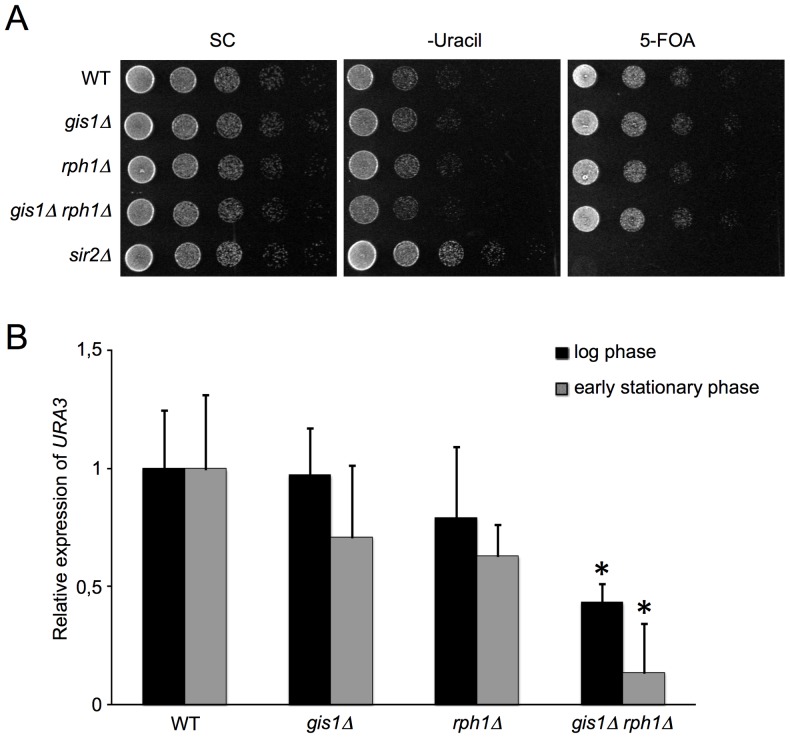
Assay for subtelomeric silencing. The expression of a subtelomerically integrated *URA3* marker was monitored in different yeast strains using spotting onto synthetic complete, uracil-less and 5-FOA-containing media (A) and qPCR (B), as described in Materials and Methods. The asterisks mark significant differences from the WT (p<0.02).

In an attempt to further analyze the role of Rph1 (and Gis1) in subtelomeric gene expression, we used a strain where a copy of the *URA3* gene has been integrated close to a telomere [52]. Expression at this locus is stochastic, with the majority of a cellular population having *URA3* silenced and, as the strain lacks the wild type *URA3* gene, expression of the reporter can be selected for on uracil-less media and against on 5-fluoroorotic acid (5-FOA). We proceeded to make single and double knockouts of *GIS1* and *RPH1* in the reporter strain. A *sir2Δ* strain was included as a control. On 5-FOA, the *sir2Δ* strain failed to grow, consistent with loss of *SIR2* causing loss of silencing, and therefore an increase in *URA3* expression. No such effect was seen in the single or double *gis1*Δ and *rph1*Δ strains (Figure 6A). However, on uracil-less media, where a reduction of the already low *URA3* expression can be detected, the *gis1*Δ *rph1*Δ strain grows more slowly than the wild type, indicating a reduced *URA3* gene expression (Figure 6A). This was confirmed by qPCR, as shown in Figure 6B; expression of the reporter was significantly reduced in the *gis1*Δ *rph1*Δ strain, and marginally reduced in both single knockout strains. The effect was most clearly seen in early stationary phase but a similar trend was seen also in the log phase (Figure 6B). The *URA3* reporter thus appears to be synergistically activated by Gis1 and Rph1. This suggests that Rph1 can affect telomeric gene expression through a mechanism that is unlikely to involve histone demethylation, since Rph1 in this case acts in parallel with Gis1, which does not have demethylase activity.

### Effects of Rph1 and Gis1 on genes involved in sporulation and meiosis

Besides its role in nutrient signaling and the diauxic shift, Gis1 is also known to be an activator of genes needed for spore wall synthesis during sporulation. Thus, the *DIT1*, *SPS100* and *SHC1* genes (whose promoters all contain STRE motifs) are induced during sporulation, and this induction is dependent on Gis1 [36], [53]. *SPS100* is also strongly induced upon a shift from 2% to 0.1% glucose, again in a Gis1-dependent manner [36]. A shift to low glucose resembles the diauxic shift, and in agreement with this we found that *SPS100* is induced during the diauxic shift. Interestingly, this induction is dependent on both Gis1 and Rph1. Thus, it is 11-fold reduced in the *gis1*Δ strain (p = 8.9e-14), 2-fold reduced in the *rph1*Δ strain (p = 1.8e-4), and 19-fold reduced in the *gis1*Δ *rph1*Δ (p = 1.5e-15). In the log phase, *DIT1*, *SPS100* and *SHC1* are instead repressed by Gis1 and Rph1 (data not shown).

Prompted by these observations, we looked at genes that are upregulated or downregulated more than 5-fold during sporulation [54] and tested if these genes, considered as groups, are affected by the *rph1* or *gis1* mutations or deletions. As seen in Table 8, we found that these genes, in particular those that are repressed, also are regulated by Rph1 and/or Gis1. In most cases, the set of sporulation-repressed genes is upregulated in the *rph1* and *gis1* strains. This is particularly evident in log phase, consistent with the fact that Gis1 and Rph1 act mainly as repressors in log phase [26] but significant effects are also seen in the PDS phase (Table 8). Rph1 has a more pronounced effect than Gis1 on these genes, and the *rph1-H235A* mutant has a more pronounced effect than the *rph1*Δ deletion, both alone and in the *gis1*Δ background (Table 8). This suggests that the demethylase activity of Rph1 is important for log phase repression of sporulation-repressed genes. We note, however, that the sporulation-repressed genes also are affected by the *gis1*Δ deletion, so the effects on these genes cannot be ascribed to demethylase-dependent regulation alone. It seems likely that the observed effects, as in the case of the telomere-associated genes, reflect two mechanisms: one involving the demethylase activity of Rph1, and one involving the function of Rph1 and Gis1 as zinc finger proteins that act on the promoters of target genes.

**Table 8:** Differential expression of sporulation-regulated genes.

	Activated during sporulation(a)		Repressed during sporulation(b)	
Log phase	Fold	p-value	Fold	p-value
*rph1-H235A* vs. WT	1.00	0.24	**↑ 1.04**	**7.0E-16**
*rph1*Δ vs. WT	**↑ 1.02**	**0.02**	**↑ 1.02**	**2.8E-07**
*gis1*Δ *rph1-H235A* vs. *gis1*Δ	**↓ 0.98**	**9.6E-03**	**↑ 1.04**	**3.8E-09**
*gis1*Δ *rph1*Δ vs. *gis1*Δ	**↑ 1.06**	**0.02**	**↑ 1.00**	**0.02**
*gis1*Δ vs. WT	**↑ 1.03**	**0.04**	**↑ 1.02**	**6.5E-05**
*gis1*Δ *rph1-H235A* vs. WT	1.01	0.74	**↑ 1.06**	**1.9E-13**
*gis1*Δ *rph1*Δ vs. WT	**↑ 1.09**	**1.8E-03**	**↑ 1.02**	**1.3E-06**

PDS phase	Fold	p-value	Fold	p-value
*rph1-H235A* vs. WT	**↑ 1.03**	**0.01**	**↑ 1.03**	**1.8E-07**
*rph1*Δ vs. WT	**↑ 1.05**	**5.4E-04**	**↑ 1.03**	**1.4E-03**
*gis1*Δ *rph1-H235A* vs. *gis1*Δ	0.98	0.21	**↑ 1.06**	**<2E-16**
*gis1*Δ *rph1*Δ vs. *gis1*Δ	1.02	0.27	**↓ 0.97**	**1.2E-08**
*gis1*Δ vs. WT	**↑ 1.03**	**4.6E-03**	1.00	0.06
*gis1*Δ *rph1-H235A* vs. WT	1.01	0.08	**↑ 1.06**	**5.6E-05**
*gis1*Δ *rph1*Δ vs. WT	**↑ 1.05**	**0.02**	**↓ 0.97**	**2.4E-06**

(a) The group of 341 genes that are upregulated 5-fold or more during sporulation [54].

(b) The group of 297 genes that are downregulated 5-fold or more during sporulation [54].

Expression of these groups of genes was tested against the rest of the genome with a Wilcoxon rank sum test. The mean fold change is shown, with p-values<0.05 highlighted in bold, and with arrows denoting up- and downregulation.

## Discussion

Rph1 is an active histone demethylase that can remove a methyl group from lysine 36 of histone H3 that has been di- and trimethylated [8]–[10], [12]. We have shown that Rph1 regulates gene expression downstream of the TOR, RAS/cAMP and Sch9 nutrient signaling pathways alongside its closest homolog, Gis1 [26]. We have now found that the catalytic activity of Rph1 is not essential for its role in nutrient signaling. Thus, deletion of *RPH1* activates and represses a large number of genes, but the vast majority of these genes are not significantly affected by the active site mutation *rph1-H235A* (Table 1). This is consistent with the finding that the expression of only 1 of 5 Rph1-regulated genes that were examined by qPCR was affected by an *rph1-H235A* mutation [55] and with findings in other organisms that some functions of JmjC proteins may be independent of the histone demethylase activity [56]–[58]. However, a closer examination revealed that the group of genes that are regulated by Rph1 also are affected by the active site mutation, though not as strongly as by the *rph1*Δ knockout (Table 2). Interestingly, this is true both for genes that are upregulated and downregulated by Rph1, though the effect is more pronounced in the latter case (Table 2). This raises the question how the same enzymatic activity can contribute to both Rph1-dependent activation and repression. If histone demethylation by Rph1 *e.g.* acts only to promote elongation by counteracting Rpd3(S)-dependent methylation, one would expect it to enhance activation but not repression. One model that is consistent with these findings is if the activity facilitates access of Rph1 to its target genes, *e.g.* by helping to clear histones from its binding sites. Alternatively, the *rph1-H235A* mutation may affect the folding or stability of Rph1 in addition to its effect on the enzymatic activity.

That being said, genes that are up- or downregulated in a *set2*Δ strain are affected in the opposite direction by the *rph1-H235A* mutation, an effect that is small for individual genes but highly significant when looking at the entire set of Set2 targets (Table 3). This suggests that Rph1 to some extent counteracts Set2-dependent histone methylation. A number of individual genes are also significantly affected by the *rph1-H235A* mutation, and are thus likely to be more strongly regulated by histone demethylation (Figure 5, Tables S1 and S2). Several of these genes are located close to telomeres, and consistent with this genes within 20 kb of a telomere, when considered as a group, are also significantly upregulated in the *rph1-H235A* mutant (Table 7). This fits logically with the fact that the subtelomeric regions are enriched for genes that are downregulated when *SET2* is deleted [18]. Similarly, we found that genes that are repressed during sporulation are upregulated in the *rph1-H235A* mutant, when considered as a group (Table 8). This suggests that the demethylase activity of Rph1 also plays a role in growth phase dependent regulation of these genes. However, as for the telomere-associated genes, the effects were small and highly significant only when the genes were considered as a group. Furthermore, both groups of genes are affected also by a deletion of *GIS1*, indicating a more complex regulation than just Rph1-dependent demethylation.

One important role of Set2 and H3-K36 methylation is activation of the Rpd3(S) complex which suppresses spurious intragenic transcription [15]–[17]. If Rph1-mediated demethylation acts to oppose Rpd3(S) activation, one would expect genes whose expression is induced or repressed upon elimination of the Rpd3(S) complex to be regulated in the opposite direction when *RPH1* is mutated or deleted, similar to the *set2*Δ effect in Table 3. This is indeed what we see; genes that are up- or downregulated in the two Rpd3(S) knockout mutants are as a group regulated in the opposite direction by Rph1 (Tables 4 and 5). However, this effect is rather weak and cannot explain the much stronger correlation between genes that are regulated by Rph1 (and Gis1) and those regulated by Rpd3, as evidenced both by gene expression (Tables 4 and 5, see also Weiner *et al.* [59]) and binding of Rpd3 to promoters (Table 6). Indeed, mimicking the effect of an *RPD3* deletion, genes regulated by subunits specific to the Rpd3(L) complex are in general repressed by Rph1 and Gis1 (Tables 4 and 5), suggesting that this complex contributes to (or counteracts) gene regulation by Rph1 and Gis1. Significantly, Rpd3(L) has been implicated in TOR signaling [49]–[50] and in Msn2- and Mns4-dependent stress signaling [51], just like Rph1 and Gis1 [26].

As noted above, the groups of genes that are regulated by Gis1 and Rph1, and in particular those that show specific regulation in the *rph1-H235A* mutant, are enriched for both telomere-associated genes and genes involved in sporulation. The former association could reflect the fact that silencing in yeast is more pronounced close to telomeres, making it easier to detect loss of silencing for such genes. As for the enrichment of genes involved in sporulation, we note that sporulation is induced by a combination of nitrogen starvation and the absence of fermentable carbon sources, conditions similar to those seen in the PDS phase. Furthermore, certain groups of genes such as those involved in stress resistance and cell wall biogenesis are expressed both during sporulation and during entry into stationary phase. A certain correlation between gene expression in the PDS phase and during sporulation is therefore to be expected. However, we note that the most pronounced correlations involve exponential phase cells and absence of expression: genes that are repressed during sporulation are thus repressed in log phase by Rph1 and Gis1. It is tempting to infer from this that Rph1 and Gis1 may be involved also in gene repression during sporulation, but this remains to be proven. Finally, we note that the sporulation-repressed genes correlate with genes downregulated in the *set2*Δ strain, but, interestingly, not with those downregulated in the *rpd3Δ* strain (Table S4). This suggests that the former group of genes is regulated by Set2 in a way that does not involve Rpd3, unlike the general Set2 targets for which the responses to *set2*Δ and *rpd3Δ* are highly correlated (Table S4).

The complex interplay between Rph1 and Gis1 deserves mentioning. First of all, it should be noted that they to a large extent act redundantly or synergistically, both in activating and repressing genes (Figure 2). However, in many cases the situation is much more intricate. For example, cluster P2 (Figure 5, Table S2) consists of genes that are upregulated in the *rph1-H235A* strain and also upregulated in *gis1*Δ, and the latter effect masks the effect of the *rph1-H235A* mutation (no further effect is seen in the *gis1Δ rph1-H235A* strain). In contrast, many of the genes in cluster P3 are upregulated when *RPH1* is either mutated or deleted, but this effect is instead blocked by the *GIS1* deletion (strains where *GIS1* is deleted behave as the wild type). What these two cases have in common is that the *rph1-H235A* mutation has no effect in the absence of *GIS1*. Even more complicated is the observed effect on telomere-proximal genes (Table 7). The expression of these genes increases additively by deletion of *RPH1* and *GIS1*, and the *rph1-H235A* mutation alone also causes an upregulation of these genes. However, the *rph1-H235A* mutation has the opposite effect in the *gis1*Δ background, where it reduces the expression of these genes (Table 7).

One possible interpretations of these genetic interactions would be if Gis1 regulates the *RPH1* gene or *vice versa*, but we have seen no evidence of this [26]. Another possibility is that the two proteins interact and thus regulate each other. Rph1 elutes from a size exclusion column as a homotetramer [10], and the yeast JmjC protein Jhd1 has also been suggested to function as an oligomer [19]. It is therefore possible that Gis1 and Rph1 could interact, perhaps forming a heterotetramer. However, we failed to detect any such interaction in a two-hybrid assay (data not shown). A third possibility is that the catalytically inactive Gis1 functions as a dominant negative regulator of Rph1 that binds to the same targets as Rph1 and thus prevents it from doing its job. In this context it is interesting to note that JmjC proteins from several organisms function in preventing spreading of silencing from heterochromatic regions [60], including the *Schizosaccharomyces pombe* protein Epe1 which has a catalytic site mutation identical to the one in Gis1 [3], [61].

In conclusion, we found that the expression of most Rph1-regulated genes, including those regulated by nutrient signaling, are not strongly affected by the *rph1-H235A* mutation, which shows that histone demethylation by Rph1 is not essential for this regulation. However, the active site mutation does have an effect on both upregulated and downregulated genes when considered as groups, which suggests that the enzymatic activity may enhance the ability of Rph1 to both activate and repress its target genes. Certain other groups of genes, in particular Set2 targets, subtelomeric genes and genes involved in sporulation, are similarly affected by the active site mutation, suggesting a role for Rph1-dependent histone demetylation in regulating these genes. A small number of genes are more strongly affected by the mutation, indicating a more pronounced role for the demethylase activity in regulating these genes.

## Materials and Methods

### Plasmids

All plasmids used are listed in Table S5. Plasmids pNN11 and pNN26 were made by cloning a fragment spanning from 622 bp upstream to 231 bp downstream of the *GIS1* open reading frame with *Bam*HI sites added to the ends into the vectors pFL38 [62] and pHR81 [63]. Plasmids pNN12, pNN13, pNN15, pNN17, pNN28, pNN29, pNN30, pNN31, pHGZ353 and pHGZ355 were then made by deleting or replacing specific parts of this wild type *GIS1* fragment using a two-step PCR with overlapping primers as described in Öyen et al. [64]. Plasmid pNN32 was made by PCR amplification of a fragment spanning from 660 bases upstream to 219 bases downstream of the *RPH1* ORF, with primers adding *Sal*I sites to the ends, which was cloned into the pCR2.1-TOPO vector (Invitrogen). The *rph1-H235A* mutation was introduced into pNN32 using the QuickChange site-directed mutagenesis kit (Stratagene), thus creating plasmid pNN34. Finally, the *rph1-H235A*-containing fragment was cut out from pNN34 and cloned into the *Sal*I site of the *URA3* vector pFL34 [62], creating pNN61. The entire *RPH1* fragment in pNN61 was sequenced to verify the H235A mutation, and to confirm the absence of any second-site mutations.

### Yeast strains

All yeast strains used are listed in Table S6. Strains with the BY4742 background (*MATα his3*-Δ*1 leu2*-Δ*0 lys2*-Δ*0 ura3*-Δ*0*) were used for all experiments, except for the analysis of the subtelomeric *URA3* reporter (see below). The wild type as well as the *gis1*Δ and *rph1*Δ single deletion strains were from the Euroscarf collection, while the *gis1*Δ *rph1*Δ double deletion strain, H1437, was made by crosses of the appropriate single deletions followed by tetrad dissection. Strains containing the *rph1-H235A* point mutation were made using the pop-in/pop-out gene replacement technique [65]. In short, yeast cells were transformed with plasmid pNN61, targeted to the *RPH1* gene by linearization with *Kpn*2I, followed by selection for growth on uracil-lacking media (pop-in). Excision of the plasmid by homologous recombination was then achieved by counterselecting against the *URA3* gene on media containing 5-fluoroorotic acid (pop-out). The replacement of the *RPH1* wild type gene with the *rph1-H235A* allele was verified by PCR and sequencing. This was done in the wild type as well as in the *gis1*Δ strain, thus creating strains H1653 and H1655, respectively. For the telomere-associated *URA3* marker expression studies we used yeast strains isogenic to YCB647 (*MATa ura3-52 his3*Δ*200 leu2*Δ*1 trp1*Δ*63 lys2*Δ*202 leu2*Δ*::TRP1 ADH4::URA3-TEL*) in which different genes had been knocked out.

### Growth conditions

Yeast cultures used for the microarray experiments were grown in triplicate in YPD media (2% glucose, 1% yeast extract, 2% peptone). Overnight pre-cultures were diluted to an OD_600_ of 0.1, and then kept in continuous log phase by repeated dilutions during 24 hours to ensure that no stationary phase transcripts remained. After the final dilution to an OD_600_ of 0.1, log phase cells were harvested after 3 h of growth (at an OD_600_ of 0.4) and PDS phase cells were harvested after 12 h of growth (at an OD_600_ of 7). Harvest was done by pelleting the cells by centrifugation for 5 min, followed by immediate freezing in liquid nitrogen. For gene expression analysis using reverse transcription PCR (Figure 1B), cells transformed with *URA3*-containing plasmids were grown in uracil-less synthetic media overnight and then diluted to an OD_600_ of 0.1 in YPD and grown for 20 h to allow the cells to pass through the diauxic transition, after which they were harvested as described above. Plasmid retention after this unselected growth in YPD was verified by plating aliquots of the cultures on synthetic plates with or without uracil. For the analysis of the effects of Gis1 overexpression (Figure 1B), cells transformed with pHR81-derived plasmids were grown to log phase in uracil-less synthetic media before being serially diluted in water and then spotted onto uracil-less and leucine-less synthetic media. For the silencing assay of the subtelomerically located *URA3* gene (Figure 6A), cells were grown to log phase in YPD before being serially diluted in water and spotted onto plates with synthetic complete, uracil-less and 5-FOA-containing media.

### RNA purification and reverse transcription PCR

RNA was prepared from frozen yeast cells using the RiboPure-Yeast kit (Ambion). For the reverse transcription PCR analysis (Figure 1B), cDNA was produced using equal amounts of RNA from each sample as template in a reaction with oligo dT primers and RevertAid H Minus reverse transcriptase (Fermentas). Expression of *SSA3* was monitored with this cDNA as template and gene-specific primers (for primer sequences, see Orzechowski Westholm et al., 2012) using the competitive PCR strategy of Siebert and Larrick [66]. Briefly, a short fragment of the *SSA3* gene harboring a deletion of 116 bp was added as an internal control at a known concentration directly into the PCR reaction. Relative expression of *SSA3* was then calculated as the ratio between DNA amplified using cDNA as template (a 340 bp product) and DNA amplified from the internal control (a 224 bp product). The amplified DNA was separated on 1.5% agarose gels.

### Microarray analysis and data processing

Purified RNA was hybridized to Affymetrix GeneChip Yeast Genome 2.0 arrays using the standard Affymetrix protocol. The raw data was processed using the Affy package [67] from Bioconductor [68]. The GCRMA normalization pipeline of Wu and Irizarry [69] was used. Because overall expression levels might differ between log phase and PDS phase, data from each time point was normalized and analyzed further separately. To identify genes that are differentially expressed in response to the *gis1*Δ, *rph1*Δ and *rph1-H235A* mutations, 10 genotype contrasts were considered at each of the two time points: *gis1*Δ vs. WT, *rph1*Δ vs. WT, *gis1*Δ *rph1*Δ vs. WT, *gis1*Δ *rph1*Δ vs. *gis1*Δ and *gis1*Δ *rph1*Δ vs. *rph1*Δ (to assess the effect of the gene knockouts), as well as *rph1-H235A* vs. WT, *rph1*Δ vs. *rph1-H235A*, *gis1*Δ *rph1-H235A* vs. WT, *gis1*Δ *rph1-H235A* vs. *gis1*Δ and *gis1*Δ *rph1*Δ vs. *gis1*Δ *rph1-H235A* (to assess the effect of the active site point mutation, both in a WT background and in a *gis1*Δ background). For each contrast, genes were tested for differential expression using a moderated t-statistic, with FDR correction to compensate for multiple hypotheses testing [70]–[71]. A gene was considered differentially expressed if its expression differed at least 1.5-fold between the two genotypes, and the FDR corrected p-value was below 0.01. This resulted in 40 lists of genes, with significantly up- and down-regulated genes for each contrast. The expression profiles of genes that were affected by the *rph1-H235A* mutation in either log phase or diauxic phase were clustered in MEV [72]–[73], using average linkage clustering and the Pearson Correlation distance metric. Microarray data are available in the ArrayExpress database (www.ebi.ac.uk/arrayexpress) under accession number E-MTAB-2147.

### Quantitative RT-PCR

Silencing at a subtelomeric locus was scored in derivatives of the YCB647 strain carrying a *URA3* reporter integrated at the subtelomeric *ADH4* locus on chromosome IV [52]. RNA was extracted from 2×10^7^ yeast cells in log phase and after 3 days of growth using RNeasy Mini kit (Qiagen). The RNA was digested with RNase-free DNase (Fermentas) and used for cDNA synthesis using SuperScript VILO cDNA Synthesis Kit (Applied Biosystems). The cDNA was subsequently used in quantitative Real-Time PCR using qPCR MasterMix Plus for SYBR® Green I (Eurogentec) and analysed on an Applied Biosystems 7500 Fast system. For confirmation of microarray data, iScript Advanced cDNA Synthesis Kit for RT-qPCR and SsoFast Eva Green Supermix were used and the analysis run on a Bio-Rad CFX96 RealTime System. At least 3 technical replicates were run for each biological replicate. Fold changes and p-values were calculated by feeding the delta–delta cycle threshold data into the same pipeline as used for the microarray data. Either the *ACT1* and *TDH3* genes encoding actin and glyceraldehyde-3-phosphate dehydrogenase were used as references. The following oligonucleotide primers were used: *ACT1* forward, 5′-TCATGAAGTGTGATGTCGATGTCC-3′, *ACT1* reverse, 5′-GAGCCAAAGCGGTGATTTCC-3′, *URA3* forward, 5′-CATCCTAGTCCTGTT-3′, *URA3* reverse, 5′-CTCCAGTAATTCCTT-3′, *TDH3* forward 5′-GTTGACGGTCCATCCCACAA-3′, *TDH3* reverse 5′-CCATACCGGTCAACTTACCTTG-3′, *COS12* forward 5′-AATAGGGATCGACAAATTCAGGTTC-3′, *COS12* reverse 5′-CGTCCTCTTCGCAATTTGAG-3′, *YOL131w* forward 5′-CGCGCAATCACAGTTAGAGCGAT-3′, *YOL131w* reverse 5′-TCCCCCTGCGATGAGCTTGGT-3′.

## Supporting Information

Table S1 **Genes differentially expressed in the log phase in response to the** ***rph1-H235A*** **mutation.** The clusters are those shown in figure 3. Significantly upregulated genes are shown in red, significantly downregulated genes in green.
(PDF)

Table S2 **Genes differentially expressed in the PDS phase in response to the** ***rph1-H235A*** **mutation.** The clusters are those shown in figure 3. Significantly upregulated genes are shown in red, significantly downregulated genes in green.
(PDF)

Table S3 **qPCR validation of array data.**
(PDF)

Table S4 **Cross-correlations between different groups of genes.** Significant overlap between groups of genes was tested with Fisher's Exact test. The number of genes in each group is shown in parenthesis, and for each comparison the number of overlapping genes is shown, with p-value for the overlap. Entries with p-value<0.01 are bolded. ^(a)^ Genes up- or downregulated at least 1.5-fold (p<0.01) in the *gis1*Δ vs. WT and/or *gis1*Δ *rph1*Δ vs. *rph1*Δ contrasts in log or PDS phase, as indicated [this study]. ^(b)^ Genes up- or downregulated at least 1.5-fold (p<0.01) in the *rph1*Δ vs. WT and/or *gis1*Δ *rph1*Δ vs. *gis1*Δ contrasts in log or PDS phase, as indicated [this study]. ^(c)^ Genes significantly up- or downregulated in *set2*Δ [18]. ^(d)^ Genes significantly up- or downregulated at least 2-fold in *rpd3Δ* [17]. ^(e)^ Genes whose promoters are enriched for Rpd3 binding at 2.5-fold or more [48]. ^(f)^ Genes whose coding regions are enriched for Rpd3 binding at 1.5-fold or more [48]. ^(g)^ Genes up- or downregulated at least 5-fold during sporulation [54]. ^(h)^ Genes located within 20 kb of a chromosomal end.
(PDF)

Table S5 **Plasmids.**
(PDF)

Table S6 **Yeast strains.**
(PDF)
