# Supplementary material for: The Histone Demethylase Activity of Rph1 is Not Essential for Its Role in the Transcriptional Response to Nutrient Signaling
Source: PLoS One. 2014 Jul 7;9(7):e95078. doi: 10.1371/journal.pone.0095078 (PMC4085034; doi:10.1371/journal.pone.0095078)
Supplement: Table S1 — Genes differentially expressed in the log phase in response to the rph1-H235A mutation. The clusters are those shown in figure 3. Significantly upregulated genes are shown in red, significantly downregulated genes in green. (PDF) [file pone.0095078.s001.pdf]

Table S1. Genes differentially expressed in the log phase in response to the *rph1-H235A* mutation.

Genetic contrast		*rph1-H235A* vs. WT		*rph1Δ* vs. WT		*gis1Δ rph1-H235A* vs. *gis1Δ*		*gis1Δ* vs. WT		*gis1Δ rph1-H235A* vs. WT		*gis1Δ rph1Δ* vs. WT	
Cluster	Gene	Fold	p-value	Fold	p-value	Fold	p-value	Fold	p-value	Fold	p-value	Fold	p-value
L1	YBL044W	1.1	0.50	0.9	0.35	**1.6**	**1.3E-04**	1.1	0.28	**1.8**	**8.4E-06**	1.0	0.90
	ECM10	**0.7**	**1.4E-04**	**0.7**	**7.6E-05**	0.8	5.7E-03	1.1	0.56	0.8	0.02	**0.6**	**3.3E-06**
	COS12	**3.8**	**1.9E-05**	**6.0**	**2.3E-07**	0.8	0.32	0.8	0.31	0.6	0.05	0.6	0.04
	YLR285C-A	0.9	0.60	0.8	0.24	**0.7**	**5.5E-03**	0.9	0.66	**0.6**	**1.9E-03**	0.7	6.6E-03
L2	*INO2*	**1.5**	**1.2E-03**	1.2	0.24	1.0	0.83	1.2	0.11	1.3	0.07	1.1	0.28
	MAL32	**1.5**	**9.2E-04**	1.2	0.07	1.0	0.89	1.3	0.05	1.3	0.04	1.5	3.6E-03
	COS8	1.7	0.04	**2.0**	**8.7E-03**	**2.2**	**5.2E-03**	0.9	0.74	2.0	0.01	**2.2**	**4.9E-03**
	HPA2	1.0	0.87	1.2	0.18	**0.6**	**6.8E-03**	1.1	0.65	0.7	0.02	**1.5**	**9.9E-03**
L3	YLR030W	**0.6**	**6.2E-03**	1.4	0.04	**0.5**	**1.5E-04**	1.3	0.11	**0.6**	**9.3E-03**	**2.4**	**2.1E-05**
	YKL071W	**0.6**	**1.8E-03**	0.7	0.02	0.7	0.02	0.8	0.22	**0.5**	**7.2E-04**	0.8	0.13
	AQY1	0.8	0.03	0.7	0.01	**0.5**	**8.2E-07**	**2.2**	**8.6E-07**	1.0	0.98	**3.5**	**3.2E-10**

The clusters are those shown in figure 3. Significantly upregulated genes are shown in red, significantly downregulated genes in green.
