# Supplementary material for: The Histone Demethylase Activity of Rph1 is Not Essential for Its Role in the Transcriptional Response to Nutrient Signaling
Source: PLoS One. 2014 Jul 7;9(7):e95078. doi: 10.1371/journal.pone.0095078 (PMC4085034; doi:10.1371/journal.pone.0095078)
Supplement: Table S2 — Genes differentially expressed in the PDS phase in response to the rph1-H235A mutation. The clusters are those shown in figure 3. Significantly upregulated genes are shown in red, significantly downregulated genes in green. (PDF) [file pone.0095078.s002.pdf]

Table S2. Genes differentially expressed in the PDS phase in response to the *rph1-H235A* mutation.

Genetic contrast		*rph1-H235A* vs. WT		*rph1Δ* vs. WT		*gis1Δ rph1-H235A* vs. *gis1Δ*		*gis1Δ* vs. WT		*gis1Δ rph1-H235A* vs. WT		*gis1Δ rph1Δ* vs.WT	
Cluster	Gene	Fold	p-value	Fold	p-value	Fold	p-value	Fold	p-value	Fold	p-value	Fold	p-value
P1	*SUL2*	**2.1**	**1.4E-03**	1.3	0.27	1.4	0.11	**2.1**	**1.1E-03**	**2.9**	**1.8E-05**	**1.8**	**5.6E-03**
	SNZ1	**0.5**	**3.8E-03**	**0.4**	**1.4E-04**	0.6	0.04	0.8	0.42	**0.5**	**6.0E-03**	**0.4**	**4.3E-05**
	SNO1	**0.4**	**4.5E-05**	**0.4**	**8.6E-05**	**0.4**	**1.8E-04**	1.0	0.94	**0.4**	**2.2E-04**	**0.5**	**1.8E-03**
	PAU24	0.7	0.03	0.7	0.09	**0.5**	**7.0E-04**	1.0	0.83	**0.5**	**1.2E-03**	0.9	0.34
	GTT2	**0.6**	**8.3E-06**	0.7	2.1E-04	**0.6**	**5.7E-06**	1.0	0.72	**0.6**	**2.4E-06**	1.0	0.79
P2	*OSW2*	**1.6**	**8.7E-03**	**2.0**	**1.1E-04**	1.3	0.11	**2.5**	**3.3E-06**	**3.3**	**7.1E-08**	**3.3**	**7.0E-08**
	PRM7	**1.7**	**1.4E-04**	**2.0**	**2.7E-06**	1.1	0.37	**2.6**	**2.1E-08**	**2.8**	**3.1E-09**	**3.5**	**1.1E-10**
	YOL131W	**2.1**	**1.5E-03**	**2.9**	**4.1E-05**	0.9	0.80	**6.5**	**5.5E-09**	**6.2**	**9.3E-09**	**10.2**	**9.5E-11**
	YNL034W	**0.6**	**4.2E-03**	0.7	0.03	0.9	0.54	1.0	0.88	0.9	0.65	1.1	0.42
	SET4	**1.9**	**2.3E-04**	**2.7**	**1.3E-06**	0.9	0.36	**3.2**	**7.5E-08**	**2.8**	**6.3E-07**	**4.1**	**3.3E-09**
	SPO22	**1.5**	**3.2E-03**	**2.5**	**4.7E-07**	0.9	0.56	**2.9**	**3.6E-08**	**2.7**	**1.3E-07**	**5.0**	**1.2E-11**
	ARP10	**1.9**	**6.7E-03**	**2.7**	**6.9E-05**	1.0	0.91	**2.4**	**3.7E-04**	**2.3**	**4.9E-04**	**3.4**	**4.7E-06**
	YDL241W	**1.5**	**3.1E-04**	**1.5**	**2.0E-04**	1.1	0.60	**1.5**	**3.4E-04**	**1.6**	**8.9E-05**	**2.0**	**1.7E-07**
	ADH7	**1.6**	**1.2E-04**	**1.6**	**1.6E-04**	1.0	0.86	**1.7**	**3.4E-05**	**1.7**	**2.1E-05**	**2.5**	**4.3E-09**
	YOL163W	**2.9**	**3.0E-03**	**3.8**	**3.8E-04**	1.0	1.00	**2.9**	**3.4E-03**	**2.9**	**3.5E-03**	**4.2**	**1.6E-04**
	YAL016C-B	**0.4**	**5.0E-03**	0.8	0.37	0.7	0.15	0.6	0.08	**0.4**	**3.0E-03**	0.6	0.13
P3	*PGU1*	0.7	0.23	**0.4**	**2.4E-03**	**0.5**	**6.2E-03**	0.8	0.35	**0.4**	**6.0E-04**	**0.3**	**5.1E-05**
	ECM10	0.8	0.02	**0.7**	**1.2E-04**	**0.5**	**2.4E-07**	0.8	4.6E-03	**0.4**	**3.7E-10**	**0.4**	**5.4E-09**
	YOR032W-A	0.8	0.26	**0.5**	**4.1E-03**	**0.4**	**6.3E-04**	**0.6**	**8.9E-03**	**0.2**	**5.8E-07**	**0.3**	**2.3E-06**
	YKL071W	0.8	0.14	0.7	0.04	**0.6**	**2.6E-03**	0.8	0.10	**0.4**	**3.6E-05**	0.7	0.02
	AAD10	**0.6**	**4.4E-04**	0.9	0.44	**0.6**	**4.9E-04**	**0.6**	**1.5E-04**	**0.4**	**1.2E-08**	**0.6**	**1.6E-04**
	YNR066C	**2.0**	**3.7E-03**	**2.1**	**2.6E-03**	0.9	0.61	1.5	0.09	1.3	0.22	1.7	0.02
	CAX4	**1.6**	**2.5E-03**	1.4	0.02	1.1	0.32	1.0	0.93	1.1	0.36	1.2	0.21
	DAL80	**1.6**	**2.1E-05**	1.3	2.6E-03	1.0	0.65	0.9	0.40	1.0	0.70	1.1	0.57
	GPM3	**1.6**	**1.4E-03**	1.4	0.02	1.0	0.76	1.1	0.35	1.2	0.22	1.2	0.18
	YHK8	**2.2**	**3.5E-03**	1.3	0.30	1.1	0.63	0.6	0.02	0.6	0.07	0.6	0.08
	PUG1	**1.6**	**2.5E-03**	**1.7**	**8.0E-04**	1.1	0.72	1.2	0.27	1.2	0.15	1.3	0.11
	YGL262W	**4.4**	**1.7E-09**	**7.7**	**3.0E-12**	1.0	0.91	0.9	0.42	0.9	0.49	0.7	0.01
	COS12	**2.4**	**1.5E-03**	**7.5**	**3.0E-08**	0.9	0.60	0.8	0.42	0.7	0.19	0.7	0.15
	YMR118C	0.7	0.16	0.9	0.59	**0.1**	**1.1E-10**	**0.01**	**5.6E-17**	**0.001**	**1.0E-20**	**0.003**	**5.0E-20**
	MSH4	**2.3**	**9.9E-04**	**2.1**	**1.8E-03**	0.9	0.73	1.7	0.02	1.6	0.04	1.5	0.09
P4	*AGA2*	**1.6**	**1.3E-03**	1.5	6.2E-03	1.0	0.88	1.3	0.04	1.3	0.06	**1.7**	**7.0E-04**
	SPS1	**1.9**	**3.2E-03**	1.6	0.02	1.0	0.87	1.5	0.05	1.6	0.03	**2.0**	**2.0E-03**
	ATF2	**1.7**	**7.5E-03**	**1.7**	**4.4E-03**	1.2	0.21	1.4	0.08	**1.7**	**5.1E-03**	**1.7**	**3.7E-03**
	YDL063C	**1.5**	**7.8E-03**	1.4	0.03	1.3	0.06	1.0	0.76	1.4	0.03	1.2	0.32
	NRP1	**1.7**	**9.9E-04**	1.3	0.05	1.1	0.40	1.2	0.31	1.3	0.07	1.1	0.34
	SLZ1	**1.5**	**4.1E-04**	1.0	0.75	1.1	0.30	1.0	0.89	1.1	0.24	1.0	0.81

The clusters are those shown in figure 3. Significantly upregulated genes are shown in red, significantly downregulated genes in green.
