# Supplementary material for: The Histone Demethylase Activity of Rph1 is Not Essential for Its Role in the Transcriptional Response to Nutrient Signaling
Source: PLoS One. 2014 Jul 7;9(7):e95078. doi: 10.1371/journal.pone.0095078 (PMC4085034; doi:10.1371/journal.pone.0095078)
Supplement: Table S3 — qPCR validation of array data. (PDF) [file pone.0095078.s003.pdf]

Table S3. qPCR validation of array data.

		rph1-H235A vs. WT		*rph1Δ* vs. WT		*gis1Δ rph1-H235A* vs. *gis1Δ*		*gis1Δ* vs. WT		*gis1Δ rph1-H235A* vs. WT		*gis1Δ rph1Δ* vs. WT	
Gene	Methid	Fold	p-value	Fold	p-value	Fold	p-value	Fold	p-value	Fold	p-value	Fold	p-value
COS12	Array	**2,4**	**1,5E-03**	**7,5**	**3,0E-08**	0,9	0,60	0,8	0,42	0,7	0,19	0,7	0,15
	qPCR	**2,6**	**6,0E-03**	1,9	0,07	1,5	0,55	1,3	0,58	1,9	0,14	0,7	0,17
YOL131w	Array	**2,1**	**1,5E-03**	**2,9**	**4,1E-05**	0,9	0,80	**6,5**	**5,5E-09**	**6,2**	**9,3E-09**	**10,2**	**9,5E-11**
	qPCR	**3,5**	**9,2E-10**	1,2	0,19	**0,4**	**1,2E-05**	**2,9**	**1,6E-08**	1,1	0,57	**3,6**	**6,5E-10**
