# Supplementary material for: The Histone Demethylase Activity of Rph1 is Not Essential for Its Role in the Transcriptional Response to Nutrient Signaling
Source: PLoS One. 2014 Jul 7;9(7):e95078. doi: 10.1371/journal.pone.0095078 (PMC4085034; doi:10.1371/journal.pone.0095078)
Supplement: Table S4 — Cross-correlations between different groups of genes. Significant overlap between groups of genes was tested with Fisher's Exact test. The number of genes in each group is shown in parenthesis, and for each comparison the number of overlapping genes is shown, with p-value for the overlap. Entries with p-value<0.01 are bolded. (a) Genes up- or downregulated at least 1.5-fold (p<0.01) in the gis1Δ vs. WT and/or gis1Δ rph1Δ vs. rph1Δ contrasts in log or PDS phase, as indicated [this study]. (b) Genes up- or downregulated at least 1.5-fold (p<0.01) in the rph1Δ vs. WT and/or gis1Δ rph1Δ vs. gis1Δ contrasts in log or PDS phase, as indicated [this study]. (c) Genes significantly up- or downregulated in set2Δ [18]. (d) Genes significantly up- or downregulated at least 2-fold in rpd3Δ [17]. (e) Genes whose promoters are enriched for Rpd3 binding at 2.5-fold or more [48]. (f) Genes whose coding regions are enriched for Rpd3 binding at 1.5-fold or more [48]. (g) Genes up- or downregulated at least 5-fold during sporulation [54]. (h) Genes located within 20 kb of a chromosomal end. (PDF) [file pone.0095078.s004.pdf]

**Table S4.** Cross-correlations between different groups of genes.

| | | gis1 log down[(a)] (29) | gis1 log up[(a)] (114) | gis1 pds down[(a)] (113) | gis1 pds up[(a)] (120) | rph1 log down[(b)] (28) | rph1 log up[(b)] (171) | rph1 pds down[(b)] (72) | rph1 pds up[(b)] (109) | set2 down[(c)] (290) | set2 up[(c)] (492) | rpd3 down[(d)] (105) | rpd3 up[(d)] (110) | Rpd3 promoter[(e)] (595) | Rpd3 ORF[(f)] (292) | Sporul. down[(g)] (297) | Sporul. up[(g)] (341) |
|---|---|---|---|---|---|---|---|---|---|---|---|---|---|---|---|---|---|
| gis1 pds down[(a)] | (113) | **13 1,7E-15** | **13 2.7E-07** | | | | | | | | | | | | | | |
| gis1 pds up[(a)] | (120) | 1 0.46 | **33 4.5E-30** | N.D. | | | | | | | | | | | | | |
| rph1 log down[(b)] | (28) | **15 1.5E-29** | 0 1.00 | **6 1.3E-05** | 0 1.00 | | | | | | | | | | | | |
| rph1 log up[(b)] | (171) | **5 1.5E-03** | **87 4.3E-119** | **24 1.0E-14** | **31 1.6E-21** | N.D. | | | | | | | | | | | |
| rph1 pds down[(b)] | (72) | **4 4.2E-04** | 4 0.05 | **28 1.8E-30** | 0 0.41 | **8 1.0E-09** | 4 0.17 | | | | | | | | | | |
| rph1 pds up[(b)] | (109) | 2 0.10 | **22 6.5E-17** | **7 5.4E-03** | **55 4.6E-68** | 0 1.00 | **26 3.3E-17** | N.D. | | | | | | | | | |
| set2 down[(c)] | (290) | 5 0.01 | **17 4.8E-05** | 6 0.83 | **18 2.7E-05** | **6 2.3E-03** | 15 0.03 | 5 0.41 | **15 3.3E-04** | | | | | | | | |
| set2 up[(c)] | (492) | 3 0.73 | **21 5.9E-04** | 11 0.61 | 13 0.33 | **7 7.8E-03** | **35 8.8E-07** | **15 9.2E-04** | 9 1.00 | N.D. | | | | | | | |
| rpd3 down[(d)] | (105) | 0 1.00 | **8 1.1E-03** | 1 0.72 | **7 6.1E-03** | 1 0.40 | 7 0.04 | 0 0.64 | **8 7.8E-04** | **13 2.2E-03** | 13 0.16 | | | | | | |
| rpd3 up[(d)] | (110) | 1 0.43 | **7 5.9E-03** | 2 1.00 | 6 0.03 | 2 0.10 | **12 9.1E-05** | 4 0.05 | **9 2.1E-04** | 1 0.04 | **24 1.3E-05** | N.D. | | | | | |
| Rpd3 promoter[(e)] | (595) | 1 0.36 | 6 0.11 | 15 0.27 | 11 0.88 | 2 1.00 | 10 0.07 | 7 1.00 | 15 0.20 | **58 1.9E-07** | **32 4.7E-03** | **3 8.3E-03** | 16 0.15 | | | | |
| Rpd3 ORF[(f)] | (292) | 2 0.66 | 6 0.83 | 4 0.66 | 3 0.29 | 1 1.00 | 7 0.72 | 2 0.59 | 1 0.04 | 17 0.49 | **13 7.16E-03** | 9 0.11 | 1 0.04 | 18 0.02 | | | |
| Sporul. down[(g)] | (297) | 2 0.66 | 3 0.29 | 10 0.08 | 9 0.21 | 0 0.40 | 3 0.03 | 2 0.59 | 7 0.51 | **49 3.7E-14** | 27 0.75 | 4 0.66 | 3 0.38 | **102 5.3E-32** | 9 0.10 | | |
| Sporul. up[(g)] | (341) | 0 0.41 | **18 1.1E-04** | 9 0.32 | **18 2.2E-04** | 3 0.23 | **22 4.6E-04** | 7 0.20 | **20 4.6E-06** | 19 0.61 | 26 0.62 | 7 0.68 | **17 2.3E-04** | 33 0.93 | 19 0.61 | N.D. | |
| Sub-telomeric[(h)] | (221) | **5 4.5E-03** | **11 4.2E-03** | 10 0.01 | **19 1.1E-07** | **5 3.8E-03** | 13 0.02 | 1 0.53 | **15 1.5E-05** | **27 2.3E-05** | 24 0.22 | **23 4.9E-12** | 0 0.02 | **2 3.3E-08** | 9 0.64 | 7 0.21 | 6 0.04 |

Significant overlap between groups of genes was tested with Fisher's Exact test. The number of genes in each group is shown in parenthesis, and for each comparison the number of overlapping genes is shown, with p-value for the overlap. Entries with p-value < 0.01 are bolded. [(a)] Genes up- or downregulated at least 1,5-fold (p<0.01) in the *gis1Δ* vs. WT and/or *gis1Δ rph1Δ* vs. *rph1Δ* contrasts in log or PDS phase, as indicated (this study). [(b)] Genes up- or downregulated at least 1,5-fold (p<0.01) in the *rph1Δ* vs. WT and/or *gis1Δ rph1Δ* vs. *gis1Δ* contrasts in log or PDS phase, as indicated (this study). [(c)] Genes significantly up- or downregulated in *set2Δ* (Tompa and Madhani, 2007). [(d)] Genes up- or downregulated at least 2-fold in *rpd3Δ* (Keogh et al., 2005). [(e)] Genes whose promoters are enriched for Rpd3 binding at 2.5-fold or more (Kurdistani et al 2002). [(f)] Genes whose coding regions are enriched for Rpd3 binding at 1.5-fold or more (Kurdistani et al 2002). [(g)] Genes up- or downregulated at least 5-fold during sporulation (Chu et al., 1998). [(h)] Genes located within 20 kb of a chromosomal end.
