# Supplementary material for: The Histone Demethylase Activity of Rph1 is Not Essential for Its Role in the Transcriptional Response to Nutrient Signaling
Source: PLoS One. 2014 Jul 7;9(7):e95078. doi: 10.1371/journal.pone.0095078 (PMC4085034; doi:10.1371/journal.pone.0095078)
Supplement: Table S5 — Plasmids. (PDF) [file pone.0095078.s005.pdf]

Table S5. Plasmids.

Plasmid	Relevant description	Source
pFL38	*CEN6 ori URA3*	Bonneaud et al. (1991)
pNN11	pFL38 - *GIS1*	This study
pNN12	pFL38 - *gis1-H204A*	This study
pNN13	pFL38 - *gis1-JmjNΔ* (aa 12-53)	This study
pNN15	pFL38 - *gis1-ZnFΔ* (aa 828-877)	This study
pNN17	pFL38 - *gis1-JmjCΔ* (aa 189-316)	This study
pHGZ353	pFL38 - *RPH1* with ZnF (aa 709-763) replaced with ZnF from *GIS1* (aa 828-882)	This study
pHGZ355	pFL38 - *GIS1* with ZnF (aa 828-882) replaced with ZnF from *RPH1* (aa 709-763)	This study
pHR81	*2-micron ori URA3 LEU2*-d	Nehlin et al. (1989)
pNN26	pHR81 - *GIS1*	This study
pNN28	pHR81 - *gis1-JmjNΔ* (aa 12-53)	This study
pNN29	pHR81 - *gis1-JmjCΔ* (aa 189-316)	This study
pNN30	pHR81 - *gis1-H204A*	This study
pNN31	pHR81 - *gis1-ZnFΔ* (aa 828-877)	This study
pCR2.1-TOPO	Cloning vector	Invitrogen
pNN32	pCR2.1-TOPO - *RPH1*	This study
pNN34	pCR2.1-TOPO - *rph1-H235A*	This study
pFL34	*URA3*	Bonneaud et al. (1991)
pNN61	pFL34 - *rph1-H235A*	This study
