# Supplementary material for: The Histone Demethylase Activity of Rph1 is Not Essential for Its Role in the Transcriptional Response to Nutrient Signaling
Source: PLoS One. 2014 Jul 7;9(7):e95078. doi: 10.1371/journal.pone.0095078 (PMC4085034; doi:10.1371/journal.pone.0095078)
Supplement: Table S6 — Yeast strains. (PDF) [file pone.0095078.s006.pdf]

Table S6. Yeast strains.

Strain	Relevant genotype	Source
BY4742	MATα *his3-Δ1 leu2-Δ0 lys2-Δ0 ura3-Δ0*	Euroscarf
Y14031	BY4742 *gis1*::kanMX4	Euroscarf
Y16165	BY4742 *rph1*::kanMX4	Euroscarf
H1437	BY4742 *gis1*::kanMX4 *rph1*::kanMX4	Orzechowski Westholm et al. (2012)
H1653	BY4742 *rph1-H235A*	This study
H1655	BY4742 *gis1*::kanMX4 *rph1-H235A*	This study
YCB647	*MATa ura3-52 his3Δ200 leu2Δ1 trp1Δ63 lys2Δ202 leu2Δ::TRP1 ADH4::URA3-TEL*	Brachmann et al. (1995)
YCB652	*MATa ura3-52 his3Δ200 leu2Δ1 trp1Δ63 lys2Δ202 sir2Δ2::TRP1 ADH4::URA3-TEL*	Brachmann et al. (1995)
H1737	YCB647 *gis1*::kanMX4	This study
H1738	YCB647 *rph1*::natMX4	This study
H1739	YCB647 *gis1*::kanMX4 *rph1*::natMX4	This study
